# Quantum Dynamics Simulations of Polariton Transport in a Bloch Surface Wave Cavity

**DOI:** 10.1002/nap2.70163

**Published:** 2026-06-14

**Authors:** Santanu Poddar, Pengfei Huo

**Affiliations:** ^1^ Department of Chemistry University of Rochester Rochester New York USA; ^2^ The Institute of Optics Hajim School of Engineering University of Rochester Rochester New York USA

**Keywords:** ballistic‐diffusive transport, BSW cavity, exciton–polariton transport, photonic crystal

## Abstract

Coupling excitons to quantized cavity photonic modes can significantly enhance the spatial and temporal range of excitonic transport. The formation of hybrid light–matter states, known as polaritons, plays a central role in this enhancement. Polaritons, owing to their photonic character, can greatly amplify coherent energy flow in excitonic systems and prolong their lifetime, and lead to ultrafast ballistic excitonic transport, which is typically diffusive outside a cavity. In Bloch surface wave (BSW) cavities, the group velocity of these polaritonic wavepackets has been theoretically predicted to approach the speed of light in the medium, though experiments suggest a renormalized value. In this work, we use quantum dynamics simulations with theoretical analysis on group velocity renormalization and wavepacket evolution to elucidate the transport dynamics in a Bloch Surface Wave Cavity. We show that polaritonic states facilitate ballistic flow directly through their photonic character, and that below a critical photonic fraction the ballistic component is suppressed—yet even then, transport remains four to eight orders of magnitude faster than purely excitonic diffusion. These results show qualitative, and in some cases near‐quantitative agreement with experimental trends, providing microscopic insight into the origin of fast transport in BSW polariton systems.

## Graphical abstract

Polariton Transport Quantum Dynamics for molecules (purple dots) collectively coupled to a Bloch Surface Wave Cavity. Lower panels show the polariton dispersion relation.

## 1 Introduction

Excitonic transport plays a central role in diverse natural and engineered systems [1, 2, 3], serving as a fundamental mechanism for energy transfer. From light harvesting in photosynthetic complexes to charge separation in optoelectronic devices [4] such as LEDs and solar cells [5, 6], the overall efficiency often hinges on the effectiveness of exciton migration. In Frenkel excitonic systems, transport typically proceeds via random hopping between localized sites—a consequence of structural and energetic disorder, thermal fluctuations, phonon interactions, and weak intermolecular electronic couplings [3, 7, 8]. Consequently, exciton motion is predominantly diffusive or subdiffusive [3, 7, 8, 9], which severely limits rapid, long‐range energy transfer and constrains the performance of next‐generation organic optoelectronic materials.

Recent experimental [10, 11, 12, 13, 14, 15, 16, 17, 18, 19] and theoretical advances [13, 20, 21, 22] have revealed that excitonic transport can be dramatically enhanced when molecular or hybrid materials are strongly coupled to confined photonic modes, forming light–matter hybrid quasiparticles known as polaritons. Cavity‐enhanced transport and other coherent phenomena in exciton–polariton systems [23] demonstrate that strong light–matter coupling can induce coherent, long‐range energy migration over macroscopic distances, through a ballistic transport behavior with mean‐square displacement (MSD) $\propto t^{2}$. Remarkably, such an ultrafast and ballistic excitonic transport has been observed in disordered organic semiconductors [10] and perovskite metasurfaces [16, 24], even at room temperature. Similar enhancements have been reported in systems coupled to surface lattice resonances [17, 25, 26] and Bloch surface waves (BSWs) [10, 15, 27, 28]. At a longer time, the ballistic transport of the polariton wavepacket gradually reduces to the diffusive one [13, 15], from MSD $\propto t^{2}$ to MSD ∝t. These findings suggest that light–matter hybridization offers a powerful route to overcome diffusive exciton motion and achieve macroscopic range for energy flow. The enhanced polariton transport behavior has also been observed in other related systems [14, 29]. Persistent coherence and long‐range energy propagation have been demonstrated across diverse platforms—from multilayer heterostructures [30, 31, 32] to large‐area 2D semiconductors [33]. Recent studies indicate that organic semiconductors can sustain microcavity‐like exciton–polaritons under ambient conditions without an external cavity [34]. In addition to enabling coherent transport, polaritonic systems have revealed a wealth of new phenomena, including polariton condensation [35, 36], superfluidity [37], quantized vortices [38], and the modification of chemical reaction pathways via vibrational strong coupling [39, 40, 41, 42].

When N excitonic modes couple to M photonic modes, the system forms M upper polaritons (UPs), M lower polaritons (LPs), and N−M excitonic dark states. Due to their partial photonic nature, polaritons are far more delocalized than bare excitons and can propagate coherently over hundreds of micrometers [33]. Even polaritons with high excitonic fractions (up to 80%) exhibit long‐range ballistic motion [13, 24], highlighting the robustness of polariton‐enabled long‐range energy transport. Moreover, the delocalization of dark states—governed by excitonic disorder and cavity Q‐factor—can significantly influence polariton velocities and overall transport efficiency [20, 21].

Theoretical and molecular quantum dynamics studies [13, 20, 22, 43, 44] indicate that enhanced transport primarily originates from the photonic component of polaritons, which promotes delocalization and sustains coherence between spatial excitonic sites, whereas elastic and inelastic scattering from static and dynamic disorder [21, 28, 45] suppresses ballistic motion through the excitonic channel. Static energetic disorder accounts for only a minor fraction of velocity renormalization, with dynamic (phonon‐assisted) disorder dominating at moderate temperatures [28, 45]. Nevertheless, long‐range coherence can persist despite such scattering [32, 43], aided by delocalization through the photonic field [14, 29]. Additionally, exciton–exciton interactions can counteract phonon‐induced decoherence, thereby stabilizing coherent transport [46, 47].

Recent advances in nanophotonics have enabled photonic architectures that support multiple exciton–polariton (EP) platforms—from Fabry–Pérot microcavities and metal–dielectric plasmonic interfaces to Bloch surface wave polaritons (BSWPs) in dielectric photonic crystals. Each platform offers distinct advantages and constraints for coherent energy transfer. In Fabry–Pérot cavities, strong coupling occurs between excitons and cavity photon modes confined between two mirrors—metallic or dielectric distributed Bragg reflectors (DBRs). Although theoretical group velocities from LP dispersion slopes can reach tens of μm⋅p$s^{-1}$, experimentally measured values are typically lower (1–2μm⋅p$s^{-1}$) [18, 48] than the theoretical value taken from the derivative of the dispersion curve. This renormalization of polaritonic group velocity arises mainly from exciton–phonon interactions and static or dynamic disorder [21, 43, 49]. A superexchange‐like mechanism between polaritonic and dark excitonic states has been proposed to explain this group velocity renormalization [49]. In addition, cavity photon loss—particularly in metallic systems—diminishes coherence and shifts transport from ballistic to diffusive [20, 21]. As the excitonic fraction increases, this diffusive transition becomes more pronounced, often erasing the ballistic character entirely.

BSW‐polaritons (BSWPs) form at the interface of a homogeneous dielectric and a periodic dielectric structure, supported by a single DBR stack. These Bloch surface waves exhibit low‐loss, evanescent propagation with nearly linear dispersion near k=0, enabling significantly higher group velocities than Fabry–Pérot or surface plasmon polariton (SPP) systems [10, 15, 27]. Without metallic components, BSWPs experience much lower photonic dissipation, achieving propagation speeds of $10^{7}$–$10^{8}$ m⋅ $s^{-1}$ [10, 15, 27], at least an order of magnitude higher than planar cavity or SPP systems ($∼10^{6}$ m$\cdot s^{-1}$ [17]). Even polaritonic states with high excitonic content (40%) can maintain ballistic propagation. When transport becomes diffusive, BSWPs still exhibit diffusion constants [15] two orders of magnitude larger than their plasmonic counterparts [17]. These characteristics make BSWPs a uniquely promising platform for studying exciton–polariton transport and its fundamental mechanisms. In an early theoretical contribution, Sokolovskii et al. [28] simulated the polariton transport quantum dynamics using an ab initio on‐the‐fly approach for methylene blue molecules coupled to the BSW cavity. They found that when only including static disorders, the MSD will not exhibit the ballistic to diffusive transition, as observed in the experiments. Only when including the nuclear fluctuations (phonons) does MSD exhibit the transition behavior observed in the experiments [15].

In this work, we aim to elucidate the transport dynamics of BSWP systems through quantum dynamics simulations with theoretical analysis on group velocity renormalization and wavepacket evolution. Specifically, we investigate how the spatial width of an excitonic wavepacket evolves when matter is coupled to BSWs under varying initial conditions. Using a mean‐field Ehrenfest approach, we simulate the propagation of lower polariton (LP) wavepackets with tunable energy composition, and extract group velocities and transport exponents as functions of photonic fraction. Our theoretical simulations reproduce the basic trends of the experimental findings in Ref. [15], with dynamical details that clarify the mechanisms governing ballistic‐to‐diffusive transport transitions. Our results in Figure 5 are also in line with ab initio MSD simulations in Ref. [28]. Building upon it, our work also extends beyond the theoretical analysis in Ref. [28], with a detailed analysis of LP polariton and exciton wavepacket (Figure 2), $v_{g}$ renormlaization as a function of excitonic contribution (Figure 4), as well as how polariton diffusion coefficients vary as a function of initial photonic contribution (Figure 6). The results show near‐quantitative agreement with experimental trends and provide valuable insights into the microscopic origin of ballistic transport in BSWP systems.

## 2 Theory

### 2.1 Hamiltonian

We consider a system composed of a one‐dimensional lattice of Frenkel excitons coupled to a BSW cavity. The states $|g_{n}\rangle$ and $|e_{n}\rangle$ represent the ground and excited states at the $n_{\text{th}}$ site, respectively. The global excitonic ground state is given by $|G\rangle =\underset{n}{⊗}|g_{n}\rangle$, and the N possible singly excited states are denoted by {$|E_{n}\rangle =|e_{n}\rangle \underset{s\neq n}{⊗}|g_{s}\rangle$}. The excitonic system is modeled using the tight‐binding approximation, whereas the light–matter interaction is described using a generalized Tavis–Cummings model [13, 50, 51, 52, 53]. Each exciton is locally coupled to a phonon bath via a Holstein‐type interaction [54].

The full Hamiltonian of our system, referred to as the generalized Holstein–Tavis–Cummings (GHTC) Hamiltonian, is given by

(1) $$\begin{array}{cc} \hat{H}= & \;\hbar \omega_{0}\sum_{n=0}^{N-1}{\hat{\sigma }}_{n}^{†}{\hat{\sigma }}_{n}+\hbar \sum_{k}\omega_{k}{\hat{a}}_{k}^{†}{\hat{a}}_{k}+{\hat{H}}_{ex-b}+{\hat{H}}_{b}\\ & +\hbar g_{c}\sum_{k,n}\left({\hat{a}}_{k}^{†}{\hat{\sigma }}_{n}e^{-ikx_{n}}+{\hat{\sigma }}_{n}^{†}{\hat{a}}_{k}e^{ikx_{n}}\right), \end{array}$$

where ${\hat{\sigma }}_{n}^{†}$ and ${\hat{\sigma }}_{n}$ are the creation and annihilation operators for an exciton at the $n_{\text{th}}$ site, defined as ${\hat{\sigma }}_{n}=|g_{n}\rangle \langle e_{n}|$. The excitation energy $\hbar \omega_{0}$ is identical at all N sites. Further, $\hbar \omega_{k}$ is the energy of the photonic mode with the in‐plane component of wave vector k (parallel to the plane of the Bloch surface wave propagation, commonly referred to as $k_{\|}$, and is considered as the x‐direction).

The operators ${\hat{a}}_{k}^{†}$ and ${\hat{a}}_{k}$ are the creation and annihilation operators for the $k_{\text{th}}$ mode, with ${\hat{a}}_{k}=|0_{k}\rangle \langle 1_{k}|$, where $|0_{k}\rangle$ and $|1_{k}\rangle$ denote the vacuum and single‐photon Fock states, respectively, within the single excitation subspace as we considered in the transport dynamics. Here, we consider the BSW cavity that has a dispersion relation as follows [15, 28, 55, 56].

(2) $$\omega_{k}=\omega_{c}+\left(\frac{c}{n_{r}}\right)|k|,$$

where c is the speed of light, $n_{r}$ is the refractive index of the medium, and |k| is the magnitude of the in‐plane component of the photonic wave vector. The constant offset $\hbar \omega_{c}$ sets the energy intercept of the approximately linear BSW branch within the spectral window of interest, that is, it fixes the reference energy at k=0 used to match the experimentally reported dispersion (see Section II in the Supporting Information S1 for a heuristic derivation.)

The second line of Equation (1) is the generalized Tavis–Cummings term describing the exciton‐photon interaction, with explicitly assuming the rotating wave approximation (that ingores counter rotating wave terms ${\hat{a}}_{k}^{†}{\hat{\sigma }}_{n}^{†}$ and, ${\hat{a}}_{k}{\hat{\sigma }}_{n}$). Further, $x_{n}$ is the center of the $n_{\text{th}}$ site, where $x_{n}=nL$. We impose periodic boundary conditions on the lattice, identifying the $N_{\text{th}}$ site with the $0_{\text{th}}$ site. The parameter $g_{c}$ denotes the single‐molecule light–matter coupling strength, which is assumed to be independent of the photonic wave vector k.

The phonon vibrations (bath) have a Hamiltonian with the following form

(3) $${\hat{H}}_{b}=\sum_{n,v}\hbar \omega_{v}\left({\hat{b}}_{n,v}^{†}{\hat{b}}_{n,v}+\frac{1}{2}\right)=\sum_{n,v}\left(\frac{{\hat{p}}_{n,v}^{2}}{2}+\frac{1}{2}\omega_{v}^{2}{\hat{q}}_{n,v}^{2}\right),$$

where ${\hat{b}}_{n,v}^{†}$ and ${\hat{b}}_{n,v}$ are the creation and annihilation operators for the $v_{\text{th}}$ phonon mode associated with the $n_{\text{th}}$ site, with vibrational frequency $\omega_{v}$. The corresponding position and momentum operators are ${\hat{q}}_{n,v}$ and ${\hat{p}}_{n,v}$, and all phonons are assumed to have the normalized mass. The exciton–phonon interaction Hamiltonian is

(4) $${\hat{H}}_{\text{ex}‐b}=\sum_{n,v}d_{v}{\hat{\sigma }}_{n}^{†}{\hat{\sigma }}_{n}\left({\hat{b}}_{n,v}^{†}+{\hat{b}}_{n,v}\right)=\sum_{n,v}\gamma_{v}{\hat{\sigma }}_{n}^{†}{\hat{\sigma }}_{n}{\hat{q}}_{n,v},$$

where $\gamma_{v}$ is the coupling constant characterizing the Holstein‐type interaction between the excitons and their local phonon environment.

We restrict our analysis to the single‐excitation subspace. The corresponding basis states include: the exciton–photon ground state $|G,0\rangle =|G\rangle \underset{k}{⊗}|0_{k}\rangle$, the photon‐dressed excitonic ground states

(5) $$|G,1_{k}\rangle =|G\rangle ⊗|1_{k}\rangle \underset{k^{\prime }\neq k}{⊗}|0_{k^{\prime }}\rangle ,$$

and the purely excitonic singly excited states with no photons

(6) $$|E_{n},0\rangle =|E_{n}\rangle {⊗}_{k}|0_{k}\rangle .$$

### 2.2 Polaritons

Polaritons arise from the superposition between exciton states and photonic excitation. In the reciprocal excitonic basis, for a given in‐plane momentum k, the two polariton states can be expressed as linear combinations of their constituent exciton and photon states [13, 57].

(7a) $$|{+}_{k}\rangle =\sin\;\phi_{k}|E_{k},0\rangle +\cos\;\phi_{k}|G,1_{k}\rangle ,$$

(7b) $$|{-}_{k}\rangle =\cos\;\phi_{k}|E_{k},0\rangle -\sin\;\phi_{k}|G,1_{k}\rangle ,$$

where $|{+}_{k}\rangle$ and $|{-}_{k}\rangle$ denote the upper polariton (UP) and lower polariton (LP) states, respectively.

The mixing angle $\phi_{k}$, which quantifies the degree of hybridization between exciton and photon modes, is defined as [50, 57]:

(8) $$\phi_{k}=\frac{1}{2}\tan^{-1}\left(\frac{2\sqrt{N}g_{c}}{\omega_{k}-\omega_{0}}\right),\;\phi_{k}\in \left[0,\frac{\pi }{2}\right].$$

$\sin\;\phi_{k}$ and $\cos\;\phi_{k}$ are called the Hopfield coefficients.

The state $|E_{k},0\rangle$ represents an exciton with in‐plane momentum k and no photon:

(9) $$|E_{k},0\rangle =\frac{1}{\sqrt{N}}\sum_{n}e^{ikx_{n}}|E_{n},0\rangle .$$

The energies of the polariton branches are given by [13]:

(10) $$\omega_{\pm ,k}=\frac{\omega_{k}+\omega_{0}}{2}\pm \frac{1}{2}\sqrt{{\left(\omega_{k}-\omega_{0}\right)}^{2}+4Ng_{c}^{2}}.$$

In this model, polaritons are formed only when the wave vector of a photonic mode matches that of a collective excitonic mode. This condition is satisfied only at a discrete set of Mk‐values corresponding to the allowed photon modes. The collection of these wave vectors is denoted as $\left\{k_{P}\right\}$. The polariton exhibits band‐like transport characterized by the group velocity [15].

(11) $$\begin{array}{cc} v_{g,\pm }\left(k\right) & =d\omega_{\pm ,k}/dk_{,}\\ & =\frac{1}{2}\left[1\pm \frac{\left(\omega_{k}-\omega_{0}\right)}{\sqrt{{\left(\omega_{k}-\omega_{0}\right)}^{2}+4Ng_{c}^{2}}}\right]\frac{d\omega_{k}}{dk}, \end{array}$$

where the k‐dependence of $\omega_{\pm k}$ is carried by the bare photonic dispersion $\omega_{k}$ as given in Equation (2). For, our BSW cavity with a linear dispersion, $\frac{d\omega_{k}}{dk}$ in Equation (11) can be replaced by $\frac{c}{n_{r}}sign\left(k\right)$. Furthermore, by following the definition of the polaritonic states according to Equations (7a) and (7b), the multiplicative prefactor in Equation (11) corresponds precisely to the photonic component of the respective UP and LP states at that particular k, $|\alpha_{ph,\pm }\left(k\right){|}^{2}$. Thus, Equation (11) simplifies to

(12) $$v_{g,\pm }\left(k\right)=\frac{c}{n_{r}}|\alpha_{ph,\pm }\left(k\right){|}^{2}\cdot sign\left(k\right).$$

Excitons with in‐plane momenta $k^{\prime }$ that do not coincide with any photonic mode of comparable energy at the same momentum remain effectively uncoupled from the light field and are referred to as *dark states*. These purely excitonic states can be written in the reciprocal basis as

(13) $$|D_{k^{\prime }}\rangle =\frac{1}{\sqrt{N}}\sum_{n}e^{ik^{\prime }x_{n}}|E_{n},0\rangle ,\;\forall \;k^{\prime }\in \left\{k_{D}\right\},$$

where $\left\{k_{D}\right\}$ denotes the set of in‐plane wave vectors for which no photonic mode is near‐resonant with the excitonic dispersion at the same momentum.

The emergence of dark excitonic states is not a finite‐size or discretization artifact, but rather a direct consequence of the momentum‐ and energy‐selective nature of exciton–photon coupling. Hybridization with the cavity field requires both momentum matching and near‐resonance between excitonic and photonic modes. Excitonic modes that satisfy these conditions form bright states and participate in polariton formation, whereas excitons that are either strongly detuned or lack a momentum‐matched photonic mode remain effectively decoupled and constitute the dark manifold. Although dark states do not directly hybridize with the light field, they play an important role in polaritonic dynamics by mediating energy redistribution and decoherence processes among the bright polaritonic modes [21, 48, 49, 58, 59, 60, 61].

Together, the upper and lower polariton states ($\left\{\pm_{k}\right\}$) and the dark excitonic states ($\left\{D_{k^{\prime }}\right\}$) form a complete set of eigenstates of the polaritonic Hamiltonian ${\hat{H}}_{\text{pl}}=\hat{H}-{\hat{H}}_{\text{ex}‐b}-{\hat{H}}_{b}$ (see Section III in the Supporting Information S1).

### 2.3 Computational Details

The system contains N=50001 sites, with an intersite spacing of L=100 Å, and includes M=4001 photonic modes spanning the energy range $\hbar \omega_{k}\in \left[0.698,3.898\right]$ eV. The intersite spacing agrees with the early experiments [48] up to the order of the magnitude (where the molecule was dissolved at 10% weight relative to the PMMA matrix, and using the concentration reported there, one estimates L=40 Å). For this reason, to a good approximation, we can ignore the intermolecular interactions due to their large separation distance in this experiment [15, 48] The total number of molecules N is chosen to ensure a large enough simulation box size (N−1)L, such that in the dynamics simulations in this work, the polariton wavepacket does not hit the boundary of the box. The number of the photon modes M was chosen such that in the range of the photonic k, the photonic energy is chosen in a range so that these cavity modes effectively couple to the excitons, with criteria $|\omega_{k}-\omega_{0}|\leq 25\cdot \sqrt{N}g_{c}$. To describe the linear dispersion of the bare BSW photonic modes, together with the excitonic and polaritonic dispersions shown in Ref. [15], we use the following parameters: the excitation energy $\hbar \omega_{0}=2.12\;\text{eV}$, the base photonic energy at the normal incidence (with k=0) with $\hbar \omega_{c}=0.698\;\text{eV}$, the refractive index $n_{r}=1.55$ and the Rabi splitting $\Omega_{R}=2\hbar \sqrt{N}g_{c}=134\;\text{meV}$. For M photonic modes, the wave vectors are discretized as $k_{j}=j\cdot \frac{2\pi }{NL}$, with $j\in \left\{-\frac{M-1}{2},-\frac{M-3}{2},\text{…},0,\text{…},\frac{M-3}{2},\frac{M-1}{2}\right\}$, where M is an odd number.

To initialize the quantum dynamics at a chosen energy $E_{0}$, the initial wavepacket is constructed as a localized linear combination of all lower polaritonic (LP) states lying energetically close to $E_{0}$. The coefficients of these individual LP states follow a Gaussian distribution centered at $k_{M}$, such that $\hbar \omega_{-,k_{M}}\approx E_{0}$. This initialization closely mirrors experimental conditions [13, 15, 47], where a highly focused pump pulse with narrow energy bandwidth and a momentum‐selective probe pulse are employed. Further details on the preparation of the initial wavefunction are provided in Supporting Information S1: Section IV A.

We use the mean‐field Ehrenfest dynamics approach to simulate polariton transport [13, 21, 46, 49, 62] in a lossless BSW cavity. In this scheme, the exciton–photon degrees of freedom (DOF) are treated quantum mechanically, whereas the phonon DOF are described classically. The phonon operators ${\hat{q}}_{n,v}$ and ${\hat{p}}_{n,v}$ are replaced by their classical counterparts $q_{n,v}$ and $p_{n,v}$ in $H_{b}$ and $H_{\text{ex}‐b}$. Each site is coupled to 20 phonon modes. To determine the phonon frequencies and coupling strengths [63], we employ a Drude–Lorentz spectral density [64, 65] with a reorganization energy λ=36meV and a characteristic frequency $\omega_{b}=6\;\text{meV}$. This was the spectral density used in the previous transport simulation work that captures the acoustic phonon (low frequency) of the materials [13, 49, 66]. Future simulations are needed to include the full spectral density that also contains high‐frequency optical phonons [62]. The initial positions and momenta of the phonons are sampled from a Wigner distribution at T=300K.

The state of the quantum subsystem is represented as

(14) $$\begin{array}{cc} |\psi \left(t\right)\rangle & =\sum_{n=0}^{N-1}c_{n}\left(t\right)|E_{n},0\rangle +\sum_{k\in \left\{k_{P}\right\}}c_{k}\left(t\right)|G,1_{k}\rangle \\ & \equiv |\psi_{\text{ex}}\left(t\right)\rangle ⊕|\psi_{\text{ph}}\left(t\right)\rangle , \end{array}$$

where $|E_{n},0\rangle$ and $|G,1_{k}\rangle$ are defined in Equations (6) and (5), respectively. This wavefunction is propagated by solving

(15) $$i\hbar \frac{\partial }{\partial t}|\psi \left(t\right)\rangle ={\hat{H}}_{Q}\left(q\left(t\right)\right)|\psi \left(t\right)\rangle ,$$

where ${\hat{H}}_{Q}=\hat{H}-{\hat{H}}_{b}$ is the quantum part of the Hamiltonian (that include excitonic and photonic DOF and exciton–phonon coupling). Chebyshev propagator [67] is employed to evolve the quantum subsystem. For a short time step δt, the position and momenta of the phonons are evolved according to the mean‐field equations of motion ${\dot{q}}_{n,v}\left(t\right)=p_{n,v}\left(t\right)$, and

(16) $$\begin{array}{cc} {\dot{p}}_{n,v}\left(t\right) & =-\langle \psi \left(t\right)|\frac{\partial \hat{H}}{\partial q_{n,v}}|\psi \left(t\right)\rangle ,\\ & =-\omega_{v}^{2}q_{n,v}\left(t\right)-\gamma_{v}\langle \psi \left(t\right)|{\hat{\sigma }}_{n}^{†}{\hat{\sigma }}_{n}|\psi \left(t\right)\rangle . \end{array}$$

The classical phonon DOFs are integrated using the Velocity Verlet scheme. Further details regarding the system propagation, including the Chebyshev propagation technique, are discussed in Supporting Information S1: Section IV C. After performing quantum dynamical simulations under various initial conditions, the position of the polaritonic wavepacket was characterized by tracking the location of its tail, denoted as $\langle {x\rangle }_{\text{ta}}$. The precise definition and procedure for determining $x_{\text{ta}}$ are provided in the Supporting Information S1: Section V B. The group velocity of the wavepacket was then evaluated as the time derivative of the tail position [13, 21, 49].

(17) $${\tilde{v}}_{g}=\frac{d\langle {x\rangle }_{\text{ta}}}{dt}.$$

Next, the mean‐square displacement (MSD) of the wavepacket was computed as

(18) $$\text{MSD}\left(t\right)=\sigma_{t}^{2}-\sigma_{0}^{2},$$

where the time‐dependent variance $\sigma_{t}^{2}$ is defined by

(19) $$\begin{array}{ll} & \sigma_{t}^{2}=\langle X^{2}\left(t\right)\rangle -\langle X{\left(t\right)\rangle }^{2}\\ & =\frac{1}{\sum_{n}P_{n}\left(t\right)}\sum_{n}x_{n}^{2}P_{n}\left(t\right)-{\left(\frac{1}{\sum_{n}P_{n}\left(t\right)}\sum_{n}x_{n}P_{n}\left(t\right)\right)}^{2}. \end{array}$$

here $P_{n}\left(t\right)$ represents the total excitation population coming from exciton and photon states at site $x_{n}$, and we introduce $|G,1_{n}\rangle =\frac{1}{\sqrt{N}}\sum_{k}e^{-ikx_{n}}|G,1_{k}\rangle$. The polariton wavefunction in Equation (14) is expressed as

(20) $$\begin{array}{cc} & |\psi \left(t\right)\rangle =\sum_{n=0}^{N-1}\psi_{\text{ex}}\left(x_{n},t\right)|E_{n},0\rangle +\psi_{\text{ph}}\left(x_{n},t\right)|G,1_{n}\rangle \\ & =\sum_{n=0}^{N-1}\left[c_{n}\left(t\right)|E_{n},0\rangle +\sum_{k}\frac{1}{\sqrt{N}}e^{ikx_{n}}c_{k}\left(t\right)|G,1_{n}\rangle \right], \end{array}$$

and the population in Equation (19) is computed given by

(21) $$\begin{array}{cc} P_{n}\left(t\right)=|\psi \left(x_{n},t\right){|}^{2} & =|\psi_{\text{ex}}\left(x_{n},t\right){|}^{2}+|\psi_{\text{ph}}\left(x_{n},t\right){|}^{2},\\ & =P_{n}^{\text{ex}}\left(t\right)+P_{n}^{\text{ph}}\left(t\right) \end{array}$$

where $\psi_{\text{ex}}\left(x_{n},t\right)=\langle E_{n},0|\psi_{\text{ex}}\left(t\right)\rangle$ and $\psi_{\text{ph}}\left(x_{n},t\right)=\langle G,1_{n}|\psi_{\text{ph}}\left(t\right)\rangle$ denote the amplitude of excitation at nth site due to exciton and photon, respectively.

The temporal evolution of MSD(t) was fitted to a power‐law dependence, $\text{MSD}\left(t\right)\propto t^{\beta }$, to extract the transport exponent β. In regimes where β=1, corresponding to diffusive transport, the MSD was further fitted as

(22) MSD(t)=2Dt

to determine the diffusion coefficient D.

## 3 Results and Discussions

We investigate the polariton transport dynamics by initially exciting on the lower polariton (LP) branch, with different choices of k (as indicated by pink open circles in Figure 1) by creating an LP wavepacket of a certain energy $E_{0}$. The photonic fraction of these states decreases from 83% (for $E_{\text{LP}}=1.97\;\text{eV}$) to 44% (for $E_{\text{LP}}=2.06\;\text{eV}$), reflecting the increasing excitonic character with a higher energy in the case of negative detuning between light and matter.

**FIGURE 1:**
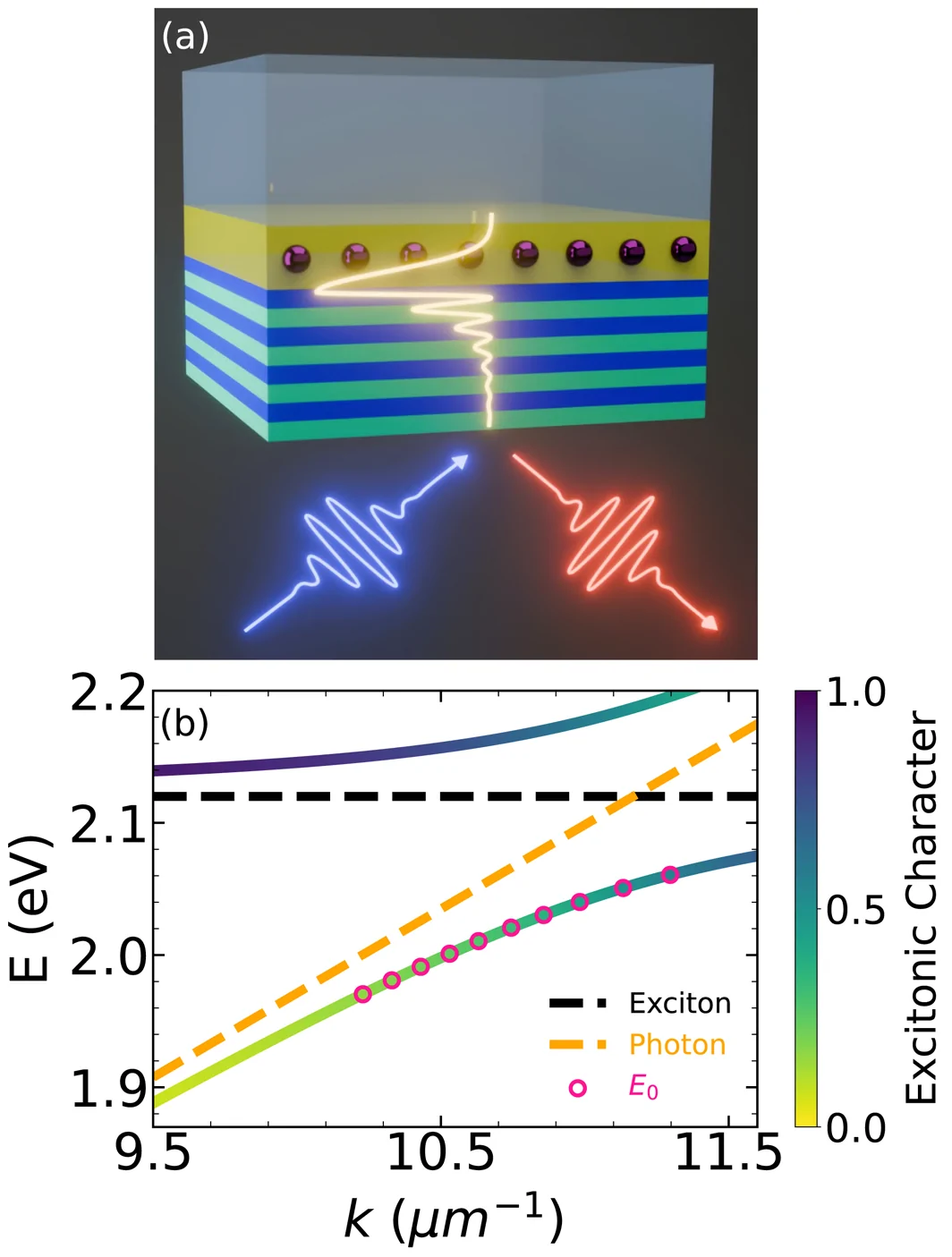
Schematic of the BSWP system and corresponding dispersion. (a) Schematic diagram of a BSWP system. The lower region consists of a Distributed Bragg Reflector (DBR) made of periodically alternating dielectric layers with two different refractive indices. The active material layer is placed on top of the DBR, whereas the upper side of the structure is in contact with air. The golden curve illustrates the electric‐field intensity profile of the BSW mode, which peaks at the DBR–active‐material interface and decays in both directions. (b) Polariton dispersion relation showing the lower (LP) and upper (UP) polariton branches. The excitonic (black dashed) and photonic (orange dashed) dispersions are plotted for reference. The color scale indicates the Excitonic character $\left(|\alpha_{\text{ex}}{|}^{2}\right)$ of the polaritonic states, ranging from photonic (yellow) to excitonic (dark violet). The pink open circles denote the location of the initial wavepacket in the LP branch for different simulations.

Figure 2a–c shows the population dynamics of excitons and photons (c.f. Equation 14) for three different initial energies, $E_{0}=1.98$, 2.00, and 2.03 eV, corresponding to excitonic fractions of 19%, 24%, and 36%, respectively. The initial fast oscillations of the populations between exciton (black) and photon (gold) are due to the light–matter coupling term (last term in Equation 1) causing the Rabi oscillation, with the frequency corresponding to the Rabi splitting. The slower transfer between exciton and photon are mainly caused by exciton–phonon coupling term (${\hat{H}}_{\text{ex}‐b}$ in Equation 4), which causes the population transfer from LP to the dark exciton manifold, and when projected to the exciton and photon basis, causes slow transitions between these two subsystems. It is evident that as the excitonic character of the initial LP state increases, the transfer of population from the photonic to the excitonic DOF (dominated by the dark exciton component) becomes faster. This behavior arises from phonon‐assisted scattering, which is more efficient for states with a larger excitonic component. The phonon scattering acts as a diabatic coupling between different polaritonic and dark states at various k, which are the eigenstates of the polaritonic Hamiltonian, ${\hat{H}}_{\text{pl}}$. Since the phonons couple directly to the excitonic part of the polaritonic states, relaxation into purely excitonic dark states proceeds more rapidly when the LP wavepacket has a stronger excitonic character.

**FIGURE 2:**
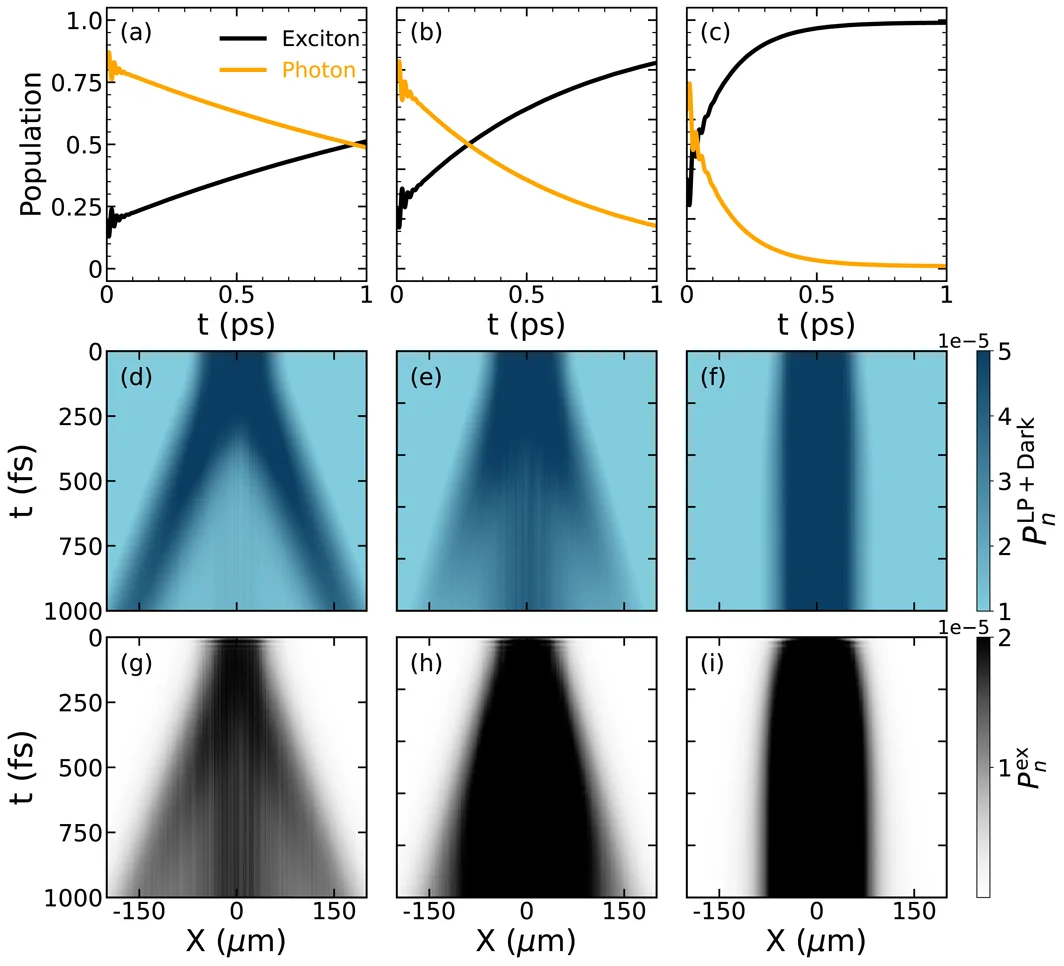
Exciton–photon population dynamics and spatiotemporal evolution of wavepackets for three different initial excitation energies $E_{0}$. Panels (a–c) display the time evolution of total excitonic and photonic populations for simulations initialized with $E_{0}=1.98$, 2.00, and 2.03 eV, respectively. Panels (d–f) and (g–i) show the corresponding spatiotemporal evolution of the lower‐polariton+Dark and excitonic populations, respectively. Although computing the site positions, the site index grid was shifted from $-\frac{N}{2}$ to $\frac{N}{2}$ such that the center of the simulation box lies at the origin. In these plots, the horizontal axis represents position along the molecular chain, the vertical axis represents propagation time, and the color scale indicates the population density.

Figure 2d–f presents the spatio‐temporal evolution of the total LP and dark‐state populations based on Equation (21), and the purely excitonic population is shown in Figure 2g–i presents the spatio‐temporal evolution of exciton component based on $P_{n}^{\text{ex}}\left(t\right)=|\psi_{\text{ex}}\left(x_{n},t\right){|}^{2}=|c_{n}\left(t\right){|}^{2}$ (c.f. Equation 14). Moving from left to right, in both the second and third rows, one observes a progressive suppression of ballistic spreading of the wavepacket and a transition toward diffusive transport as the initial LP wavepacket becomes more excitonic. In all cases, the initial wave packet originates from the center of the simulation box and propagates toward both edges.

Comparing the spatial evolution of the combined LP+dark population with that of the purely excitonic population for a given $E_{0}$, it becomes evident that the excitonic component propagates more slowly than the LP+dark wavepacket. Moreover, the excitonic population consistently retains a nonnegligible density near the center of the simulation box—a feature that is largely absent in the more photonic LP wavepackets. Since dark states are purely excitonic, they propagate very slowly (in this model, they possess no net group velocity on their own owing to the flat dispersion of the exciton branch), whereas LP states retain a ballistic component of transport depending on their photonic fraction. Consequently, the more photonic the LP state, the weaker the influence of phonon‐assisted scattering and relaxation into dark states, leading to a more pronounced ballistic transport. Conversely, for LP states with larger excitonic character, phonon‐assisted relaxation into dark states is more efficient, enhancing diffusive behavior.

The excitonic wavepacket thus arises from two contributions: a ballistic component from the LP states with finite group velocity, which is partly excitonic, and a slow moving component from the dark states that spreads only slowly, but is purely excitonic. When the initial LP wavepacket is highly photonic, its weak coupling to dark states limits population transfer, making the LP contribution dominant. In contrast, for LP wavepackets with a larger excitonic character, phonon‐mediated relaxation into dark states is faster, and these dark states—localized closer to the center—contribute most significantly to the excitonic population dynamics. The dark excitons also exhibit limited spatial propagation due to continuous population transfer from the fast‐moving LP wavepacket, a mechanism where LP transport effectively “drags” the dark‐state population, as discussed in our previous work [21]. This interplay explains why the excitonic wavepacket moves diffusively with a nonzero tail velocity even when it has a flat dispersion, but more slowly than the combined LP+dark population, and why it consistently exhibits a nonnegligible density near the center of the box.

These two competing propagation mechanisms—ballistic motion with high velocity and diffusive or, subdiffusive motion with low velocity—occur simultaneously, and the process by which the ballistic LP states dynamically “drag” the dark states is further illustrated in Figure 3. Figure 3a shows the temporal evolution of the LP, UP, and dark‐state populations for an initial energy of $E_{0}=2.00$ eV. The dynamics are dominated by population transfer from the LP to the dark manifold, whereas the LP‐to‐UP transfer channel remains negligible, due to both the large energy separation between the LP and UP branches [66, 68], as well as the low phonon‐mediated scattering efficiency in the case of the predominantly photonic UP states.

**FIGURE 3:**
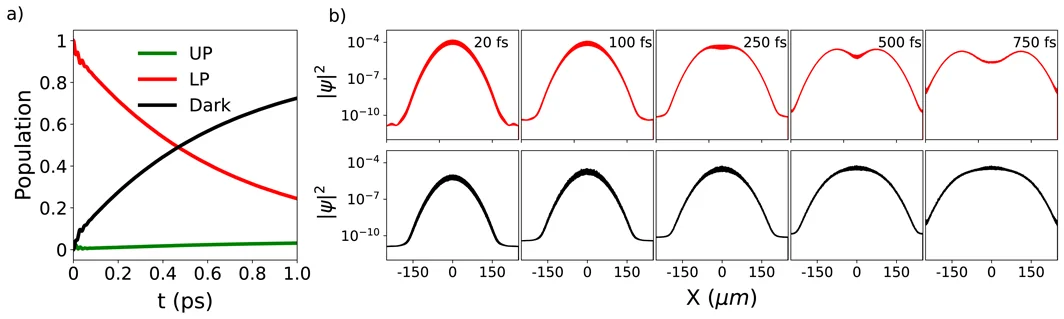
(a) Temporal evolution of lower polariton (LP), upper polariton (UP), and dark‐state populations for an initial energy of $E_{0}=2.00$ eV. (b) Site‐resolved population distributions at selected times. The top row shows contributions from LP states, whereas the bottom row displays contributions from dark states, both plotted on a logarithmic scale. Each column corresponds to a different time snapshot.

Figure 3b presents the site‐resolved population distributions of LP and dark states at selected times, clearly highlighting the contrast in their transport behavior. The LP wavepacket splits into two symmetric components, corresponding to positive‐ and negative‐momentum contributions, and rapidly propagates away from the center of the simulation box in a ballistic fashion. In contrast, the dark‐state population exhibits purely diffusive broadening: its profile gradually widens over time, but the peak remains localized near the center. This behavior arises because the dark states do not possess intrinsic group velocity in our model; they gain population through phonon‐assisted transfer from the propagating LPs and, once populated, remain nearly stationary on the timescale of observation. This mechanism explains the persistent high dark‐state population around X=0 and encapsulates the coexistence of ballistic polaritonic motion with diffusive dark‐state spreading.

Figure 4a compares the simulated group velocities (${\tilde{v}}_{g,-}$, pink open circles with dashed line) of LP wave packets at different energies with their corresponding theoretical values ($v_{g,-}$, solid line color‐coded by excitonic fraction), calculated using Equation (12). Across much of the LP branch, the theoretical group velocity approaches the speed of light, suggesting that—if preserved—such polaritons could enable ultrafast excitonic transport. However, the simulations reveal a systematic renormalization of $v_{g,-}$, even for highly photonic wave packets, with the effect becoming more pronounced as the excitonic character increases.

**FIGURE 4:**
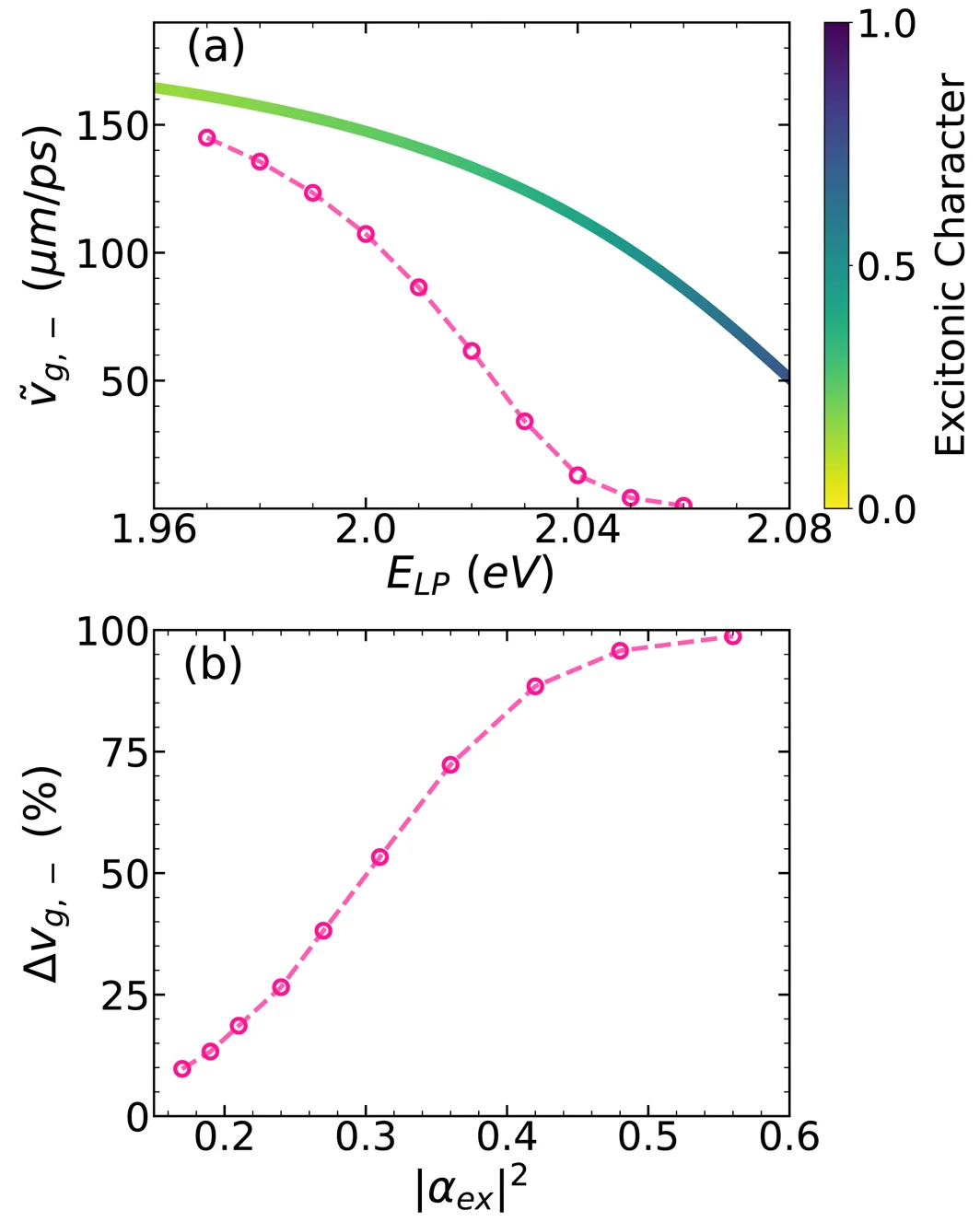
(a) Group velocity as a function of lower polariton (LP) energy, $E_{\text{LP}}$. The solid line represents the theoretical group velocity, obtained as the slope of the LP dispersion ($\partial \omega_{-,k}/\partial k$), with the color scale indicating the excitonic fraction of the polariton. Pink open circles connected by a light pink dashed line denote the renormalized group velocities extracted from simulations at different $E_{\text{LP}}$. (b) The percentage renormalization of the group velocity plotted against the excitonic character of the initial wavepacket.

Figure 4b quantifies this effect by plotting the relative renormalization, $\Delta v_{g,-}=1-{\tilde{v}}_{g,-}/v_{g,-}$, as a function of excitonic fraction. The dependence exhibits a sigmoidal trend: $\Delta v_{g,-}$ initially shows negligible renormalization till around 20% excitonic character, then grows nearly linearly, and finally saturates near 100% once the excitonic character exceeds ∼50%. Thus, even wave packets with significant photonic content can have diffusive transport with vanishing group velocity in this regime.

This renormalization originates from phonon‐assisted scattering between LP and dark states [49]. Since the scattering rate scales with the excitonic Hopfield coefficient of the LP state, larger excitonic fractions enhance the coupling and, consequently, the renormalization. Previous studies have shown that increasing phonon coupling strength—through higher reorganization energy λ, bath frequency $\omega_{f}$, or temperature T—further amplifies this effect [21]. From a theoretical perspective, the phenomenon can be interpreted either as a phonon‐induced renormalization of the polariton dispersion band via dark‐state superexchange [49], or within the polaron–polariton framework using a Floquet formalism for phonon [62], where phonons generate additional subbands between LP and UP. Both viewpoints underscore that stronger excitonic character yields stronger LP–dark coupling, driving the observed suppression of group velocity. It is worth noting that some of the previous studies have also reported phonon‐assisted enhancement of polariton group velocity, either with the presence of Peierls‐type phonons [69] or when probing LP states with a very high excitonic character (∼90%) [70]. Although such scenarios highlight the diversity of phonon–polaritonic interactions and merit further investigation, they lie beyond the scope of the present work.

We next examine the spatial spreading of polaritonic wave packets by analyzing the time evolution of the mean square displacement (MSD), defined as $\sigma_{t}^{2}-\sigma_{0}^{2}$, for wavepackets with various initial conditions. The colored open circles in Figure 5a shows how MSD of the different LP wavepackets with a varying initial photon character evolves as a function of time. Both axes are in log scale. We know that, for ballistic transport, which a purely photonic wave would undergo, the increase in MSD is proportional to $t^{2}$, whereas for diffusive transport, it is proportional to t. We have fitted the MSD of the wavepackets with $D_{\beta }t^{\beta }$ with β=1,2 in the time range of 20fs to 500fs and extracted the transport exponent β for the best fit. The fitted line is also shown in the figure. The region where MSD is proportional to $t^{2}$ is shown with a solid black line, whereas the region where MSD is proportional to t is shown with a solid red fitted line. It is evident that LP wavepackets with very high photonic character (>73%) show only ballistic transport (β=2) for almost the whole range of the fit. Lowering the photon character of the wavepacket leads to ballistic transport at initial times of the simulation and then a transition to diffusive transport at later times. On the contrary, LP wavepackets with 40% or more excitonic character show only linear temporal evolution of MSD, that is, only diffusive transport, without any ballistic signature.

**FIGURE 5:**
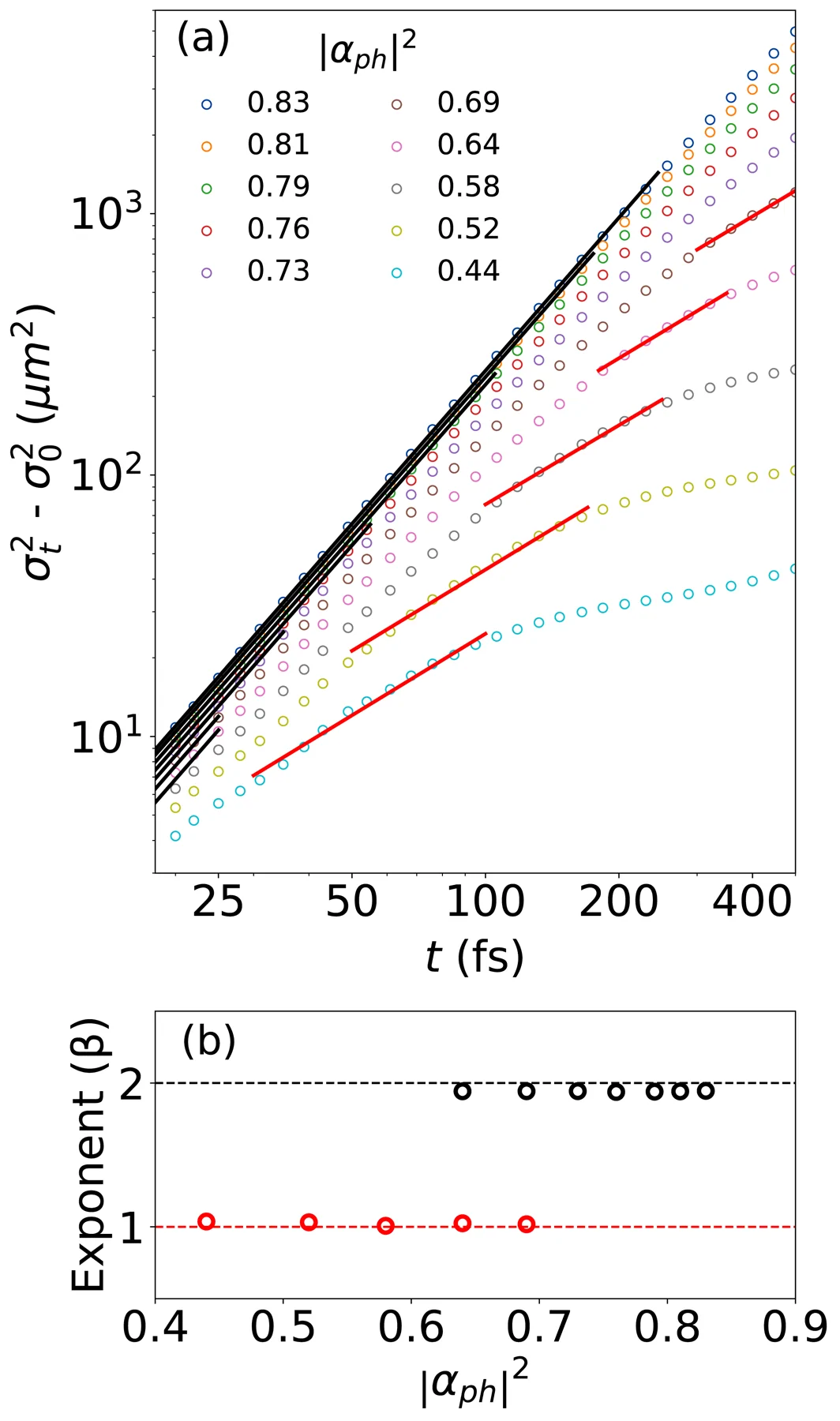
Polaritonic transport dynamics for various initial excitation energies $E_{0}$. (a) Time evolution of the mean squared displacement (MSD, $\sigma_{t}^{2}-\sigma_{0}^{2}$) for $E_{0}=1.97$–2.06 eV. Each dataset represents an initial wavepacket at a given $E_{0}$, colored by its photonic fraction. Black and red lines denote ballistic ($MSD\propto t^{2}$, β=2) and diffusive (MSD∝t, β=1) regimes, respectively. (b) Extracted transport exponent β versus photonic fraction of the initial wavepacket. Polaritonic states with a high photonic contribution (>73%) remain ballistic, and those with a low photonic contribution (<58%) are diffusive, and intermediate ones show a ballistic–diffusive crossover.

These trends are summarized in Figure 5b, which shows the extracted transport exponent β as a function of the initial photonic fraction. The systematic reduction of β with decreasing photonic character, together with the clear ballistic–to–diffusive crossover observed in several cases, highlights the crucial role of exciton–phonon scattering in suppressing ballistic propagation and driving the onset of diffusion. Notably, our theoretical results show excellent agreement with the experimental measurements reported in Figure 4 of Ref. [15].

Finally, we evaluate the diffusion coefficient D for those LP wavepackets that exhibit diffusive transport, either throughout or during a portion of the simulation time, according to the relation $\sigma_{t}^{2}-\sigma_{0}^{2}=2Dt$. The extracted D values are plotted in Figure 6 as a function of the photonic character of the initial LP wavepacket. The red open circles represent the diffusion coefficients obtained by fitting the MSD in the time window where the transport exponent β≈1 (see Equation 22), corresponding to the red solid line region in Figure 5a. For comparison, the black open circles denote the previously calculated renormalized group velocities (${\tilde{v}}_{g,-}$) for those cases exhibiting ballistic transport, whereas the solid black line indicates the theoretical LP group velocity ($v_{g,-}$) derived from the slope of the LP dispersion.

**FIGURE 6:**
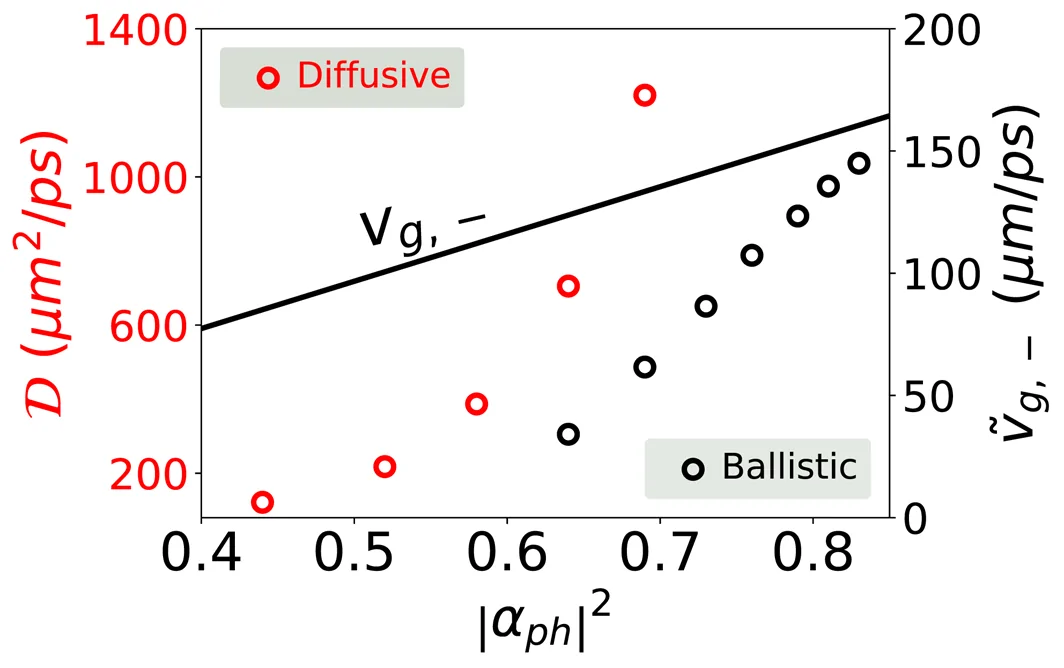
Transport characteristics as a function of the photonic fraction ($|\alpha_{\text{ph}}{|}^{2}$) of the initial state. The red open circles correspond to the diffusion coefficient D (left axis) extracted from cases where MSD scales linearly with time (MSD∝t), indicating diffusive transport. The black open circles represent the calculated group velocity ${\tilde{v}}_{g}$ (right axis) obtained for cases exhibiting ballistic transport, where $MSD\propto t^{2}$. The solid black line denotes the theoretical group velocity $v_{g}$ extracted from the polaritonic dispersion relation.

As shown in Figure 6, the diffusion coefficient D increases approximately quadratically with the photonic fraction of the initial LP wavepacket, reaching values as high as $∼1400\;\mu m^{2}\cdot {\text{ps}}^{-1}$ for highly photonic LPs. Even for LP wavepackets with a modest photonic content (≈40%), D remains remarkably large ($∼30\;\mu m^{2}\cdot {\text{ps}}^{-1}$), much larger than the typical excitonic diffusion coefficients (in the range of $10^{-3}$–$10^{-7}\;\mu m^{2}\cdot {\text{ps}}^{-1}$ in conventional semiconductors) [71, 72, 73, 74]. This is an intermediate regime (for $0.4<|\alpha_{\text{ph}}{|}^{2}<0.6$) where the polariton transport still occurs, but it is no longer ballistic. Yet, D is much larger than the purely excitonic component due to the nonzero photonic contribution.

To analyze the transition from ballistic to diffusive transport, and to clarify why the extracted diffusion coefficients remain large even for wavepackets with moderate (or even relatively low) photonic content, we evaluated the time‐dependent MSD in successive time segments using

(23) $$MSD\left(t\right)=D\left(t\right)\cdot t^{\beta \left(t\right)},$$

and computed the instantaneous transport exponent from the slope of the lnMSD(t)–lnt curve

(24) $$\beta \left(t\right)=\frac{d\;\ln MSD\left(t\right)}{d\;\ln\;t}.$$

Figure 7 summarizes the temporal evolution of β(t), the photonic population, and the polaritonic purity for a range of initial conditions (i.e., different initial LP wavepackets). As shown in Figure 7a, β(t) typically starts from a large value (around 2), undergoes a short‐time transient with oscillations, and then decays toward smaller values (close to zero). The decay rate depends strongly on the initial condition: the wavepacket that is initially more photonic maintains a larger β(t) value for longer times, whereas more excitonic initial states exhibit a faster decay toward the diffusive and subdiffusive regimes.

**FIGURE 7:**
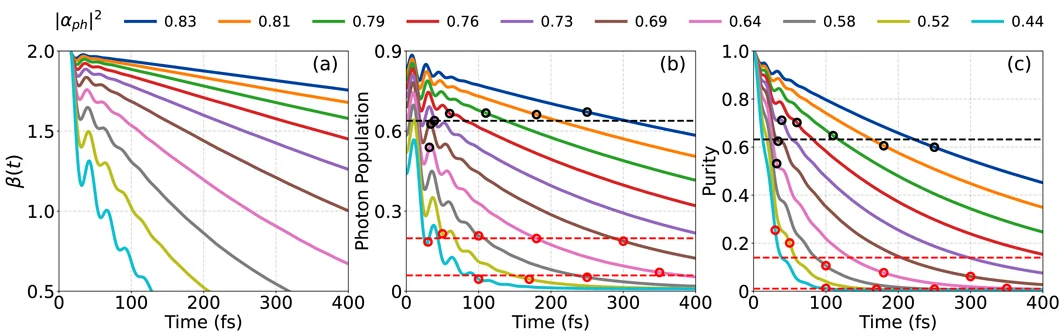
Time‐resolved transport characteristics for LP wavepackets with different initial photonic fractions $|\alpha_{\text{ph}}{|}^{2}$ (values indicated at the top). (a) Instantaneous transport exponent $\beta \left(t\right)=\frac{d\;\ln MSD\left(t\right)}{d\;\ln\;t}$ extracted from the local slope of the log–log MSD curve. (b) Corresponding photonic population as a function of time. (c) Purity $Tr\left[\rho^{2}\left(t\right)\right]$ as a measure of polaritonic coherence, where ρ is the reduced density matrix of the polaritonic system. In panels (b) and (c), black open circles denote the time at which ballistic transport ($MSD\propto t^{2}$) terminates for cases exhibiting a quadratic regime, whereas red open circles mark the onset (upper) and termination (lower) of diffusive transport (MSD∝t). The black dashed horizontal lines indicate the average values at which ballistic transport ceases (photonic population ≈64%, purity ≈0.63), and the red dashed lines mark the average photonic populations (≈20% and ≈6%) and purities (≈0.14 and ≈0.01) corresponding to the onset and end of diffusion, respectively. The consistent alignment of these crossover points across different initial conditions highlights the strong correlation between instantaneous photonic population, coherence, and the transition from ballistic to diffusive and eventually subdiffusive transport.

Here, it is important to emphasize a key modeling aspect of the present GHTC Hamiltonian: the excitonic subsystem has no nearest‐neighbor transfer integral. Consequently, the bare exciton band is flat and, in the absence of the cavity coupling, excitons have no intrinsic mobility in our model (with zero dispersion and group velocity). This is explicitly verified by performing simulations of the purely excitonic system (same exciton–phonon parameters but no light–matter coupling), reported in Supporting Information S1: Section IV, where MSD(t) remains essentially unchanged. Therefore, in the present model, all mobility originates from the photonic subsystem and is imparted to the excitonic degrees of freedom through polaritonic hybridization. From this perspective, the instantaneous growth rate of MSD(t) (and hence β(t)) is naturally expected to correlate with the instantaneous photonic population.

This correlation is further verified in Figure 7b, which shows the photonic population as a function of time for the same set of simulations. For all initial conditions, the photonic population exhibits an initial transient oscillation (including oscillations associated with polaritonic dynamics) followed by an overall decay due to phonon‐assisted scattering, from the hybrid polaritonic states into the purely excitonic dark‐state manifold. This decay rate increases for wavepackets with an increasing excitonic fraction, consistent with the hypothesis that a stronger exciton–phonon scattering through the excitonic Hopfield component is the cause for the ballistic to diffusive transition.

To make the connection between different transport regimes and instantaneous photonic population more quantitatively, we annotate Figure 7b as follows. For those initial conditions that exhibit a ballistic regime in the MSD (i.e., a $t^{2}$ segment identified by the black fitting line in Figure 5a), we mark the end of the ballistic fitting window by black open circles and record the corresponding photonic population at those times. Interestingly, across all such initial conditions, ballistic transport ceases when the photonic population drops below a certain range, with an average value of ≈64% (black horizontal dotted line). Moreover, the case whose initial photonic population is already below this value (e.g., the wavepacket starting at ∼58% photonic fraction) does not show any clear $MSD\propto t^{2}$ regime at any point in the dynamics. Together, these observations indicate that a sufficiently large photonic population is a necessary condition for sustaining ballistic propagation in this system, and that the loss of ballistic behavior during the dynamics is tightly linked to the depletion of the photonic component due to scattering to the dark states.

We carry out the same analysis for the diffusive regime. For initial conditions that display an extended window with MSD∝t (red fitting segments in Figure 5a), we mark (i) the onset of diffusion and (ii) the end of diffusion using red open circles, and record the corresponding photonic populations at those times. Remarkably, both the onset and termination of diffusion are highly consistent across different simulations when viewed in terms of the instantaneous photonic population. Averaging over all cases yields a photonic population of ≈20% at the onset of diffusion (upper red dotted line) and ≈6% at the end of diffusion (lower red dotted line). Below this latter threshold, the system enters the subdiffusive regime with β(t)<1, consistent with the long‐time bending observed in the MSD curves (Figure 5a). Importantly, the crossover from ballistic to diffusive transport is not abrupt in time: the photonic population decreases continuously, and correspondingly β(t) evolves smoothly from 2 to 1. In the intermediate window where the photonic population decreases from ∼64% to ∼20%, β(t)∈(1,2), indicating a superdiffusive regime that bridges ballistic and diffusive transport.

This photonic‐population‐based interpretation also provides a natural explanation for why the extracted diffusion constants remain orders of magnitude larger than purely excitonic diffusion. In our model, diffusion in the polaritonic system is not driven by excitonic hopping (which is absent), but rather emerges from a photonic carrier that is intrinsically ballistic and becomes progressively slowed by coupling to immobile excitonic degrees of freedom. Thus, even when the dynamics appear diffusive in terms of MSD(t)∝t, the effective carrier retains a photonic contribution and therefore produces a much larger D than a purely excitonic system would.

Several prior studies [13, 30] have argued that the ballistic‐to‐diffusive crossover in polaritonic transport is controlled by the extent of quantum coherence, and have used coherence measures to rationalize the crossover semiquantitatively. To examine this possibility in our system, we compute the purity, $Tr\left[\rho^{2}\left(t\right)\right]$, as shown in Figure 7c, with ρ(t) being the trajectory‐averaged reduced density matrix for the exciton–photon system at time t. The purity ranges from 1 (fully coherent pure state) to 1/(N+M) (maximally mixed state in the relevant subspace), and its decay reflects the loss of coherence, induced by the phonon bath in our system. The purity exhibits qualitative similarity to both β(t) (see Figure 7a) and the photonic population (see Figure 7b), all of which have an initial transient oscillations followed by a decay. We therefore repeat the same crossover‐marking procedure used for the photonic population. The end of the ballistic regime (from Figure 5a) is marked by black open circles in Figure 7c, yielding an average purity of ≈0.63 at ballistic termination (black dashed line). However, unlike the photonic population, the purity values at this crossover show noticeably larger spread across initial conditions (ranging from ∼0.53 to ∼0.71). Similarly, the onset of diffusion corresponds on average to a purity of ≈0.14 (upper red dashed line), but again with substantial variation across simulations (roughly ∼0.06 to ∼0.25). The end of diffusion occurs at a much smaller purity, with an average value ≈0.01 (lower red dashed line), which is comparatively consistent across conditions and coincides with the transition into the subdiffusive regime.

A plausible reason for the weaker universality of using purity thresholds to interpret the ballistic‐to‐diffusion transition is that all simulations begin from a pure state with $Tr\left[\rho^{2}\left(t=0\right)\right]=1$ (see Supporting Information S1: Equation S31), whereas the initial photonic population differs substantially across initial conditions. This makes the photonic population a more direct discriminator of which initial states can sustain ballistic transport in the first place and when that ballistic component collapses during the dynamics. At the same time, we emphasize that coherence and photonic population are not independent: both are influenced by phonon‐induced scattering and by the evolving polaritonic composition. Therefore, we do not conclude that coherence is irrelevant; rather, within the present model and initialization protocol, the instantaneous photonic population provides a more consistent and predictive marker for the transport‐regime crossovers than purity alone.

A more complete mechanistic understanding of how coherence, polaritonic composition, and transport regime are intertwined will require further theoretical and computational investigation, particularly in extended models that include (i) nonzero exciton hopping, so that excitons are not strictly immobile, and (ii) finite cavity loss, which introduces an additional photonic decay channel beyond phonon‐assisted transfer to the dark excitonic manifold.

These results highlight the dramatic enhancement of transport efficiency enabled by polariton formation and the key role of photonic contribution in governing polariton mobility. Although the diffusion coefficients extracted from our simulations are approximately four times larger than the experimental values reported in Ref. [15], the results in Figure 6 reproduce the same qualitative dependence (approximately quadratic) on $|\alpha_{\text{ph}}{|}^{2}$ observed experimentally (see Figure 5 in Ref. [15]). Moreover, the group velocities obtained from our simulations also show good qualitative agreement with the experimentally measured trends reported in Ref. [15]. In addition, the time‐resolved analysis of the instantaneous transport exponent reveals that the crossover from ballistic to diffusive and subsequently to subdiffusive transport is strongly correlated with the instantaneous photonic population rather than the purity of the wavepacket in our system. The emergence and termination of distinct transport regimes occur at nearly universal threshold values of photonic fraction across different initial conditions, providing a unified microscopic interpretation of the transport transitions.

## 4 Conclusion

In summary, our study establishes a comprehensive microscopic picture of phonon‐influenced polariton transport in BSW cavity, revealing how the interplay between photonic and excitonic characters governs the transition of the transport dynamics from the ballistic regime to the diffusive regime. By initializing lower polariton (LP) wavepackets with systematically varied excitonic fractions, we demonstrate in Figure 2 that phonon‐mediated coupling to dark excitonic states leads to a rapid redistribution of population and a concomitant suppression of spatial spreading of the wavepacket, which in turn results in a lowering of group velocity (Figure 4). The difference in propagation mechanism for LP and dark and how the LP “drags” the diffusively spreading dark states are apparent in Figure 3. This manifests as a continuous crossover—from purely ballistic transport for highly photonic LPs to entirely diffusive motion for strongly excitonic ones, captured quantitatively by the transport exponent β in Figure 5 and the renormalized group velocity ${\tilde{v}}_{g,-}$ highlighted in Figure 4. Beyond this global characterization, our time‐resolved analysis further reveals that the transition between transport regimes is not abrupt but governed by nearly universal threshold values of the instantaneous photonic population. In particular, ballistic transport consistently ceases when the photonic fraction decays to ∼64%, diffusion emerges when it falls to ∼20%, and subdiffusive behavior sets in below ∼6%, largely independent of the initial state. A similar, though less sharply defined, correspondence is observed with purity, indicating that instantaneous photonic population serves as a more robust predictor of transport regime across different initial conditions.

The diffusion coefficients extracted from the time‐dependent mean square displacements reveal transport efficiencies up to eight orders of magnitude higher than those of bare excitons, underscoring the profound enhancement of energy mobility enabled by light–matter hybridization. At the same time, our results highlight that the mechanism facilitating long‐range coherent transport—the photonic admixture—simultaneously reduces the excitonic participation that is crucial for device functionality. Thus, for polaritonic transport to be viable in functional optoelectronic or energy‐harvesting devices, a fundamental challenge arises: to maintain ballistic propagation while preserving a sufficiently high excitonic fraction to ensure that a significant portion of the excitation is effectively transported.

The duality of ballistic and diffusive channels uncovered here provides a unifying framework for understanding phonon‐assisted relaxation and transport in polaritonic systems. It suggests that future efforts toward practical polariton‐based devices must navigate this delicate trade‐off, potentially through tailored detuning, engineered phonon environments, or cavity designs that optimize both coherence and exciton participation. Our identification of photonic‐population‐controlled transport thresholds offers a concrete microscopic design principle: by stabilizing the photonic fraction above the ballistic threshold while mitigating rapid phonon‐induced dark‐state scattering, one may extend the temporal window of ultrafast transport without completely sacrificing excitonic functionality. Our findings, therefore, lay the groundwork for the rational control of hybrid light–matter transport in solid‐state and molecular platforms, bridging the microscopic mechanisms of polariton–phonon interactions with macroscopic functionality.

## Supporting information


Supporting Information S1

## Data Availability

The data that support the plots within this paper and other findings of this study are available from the corresponding authors upon a reasonable request.
