# Supplementary material for: Quantum Dynamics Simulations of Polariton Transport in a Bloch Surface Wave Cavity
Source: Nanophotonics. 2026 Jun 14;15(12):e70163. doi: 10.1002/nap2.70163 (PMC13642521; doi:10.1002/nap2.70163)
Supplement: Supplementary file 1 [file NAP2-15-e70163-s001.pdf]

Supplementary Material for Quantum Dynamics Simulations of Polariton Transport in a Block Surface Wave Cavity

Santanu Poddar[1,*] and Pengfei Huo[1,2,†]

[1] *Department of Chemistry, University of Rochester, 120 Trustee Road, Rochester, New York 14627, USA*

[2] *Institute of Optics, Hajim School of Engineering, University of Rochester, Rochester, New York 14627, USA*

CONTENTS

I. Details of the Model Hamiltonian S1
A. Generalized Holstein-Tavis-Cummings Hamiltonian S1
B. Model Parameters S3

II. BSW Dispersion S4

III. Polaritons and Dark States S4

IV. Details of the Theoretical Simulations S5
A. Preparation of initial quantum State S5
B. Sampling initial Phonon positions and momenta S7
C. Ehrenfest Propagation Scheme Using Chebyshev Algorithm S7

V. Calculation of Transport Properties S10
A. Spatial Distribution of the Polariton Wavepacket S10
B. Calculations of Group Velocity S11
C. Calculation of MSD S12
D. Extraction of the diffusion constant S12

VI. Simulating a Purely Excitonic System with a Phonon Bath S13

References S14

I. DETAILS OF THE MODEL HAMILTONIAN

A. Generalized Holstein-Tavis-Cummings Hamiltonian

We consider a one-dimensional (1D) lattice of excitons coupled to the electromagnetic field of a Bloch Surface Wave (BSW) cavity. The excitons are modeled using a tight-binding Hamiltonian, where each exciton interacts with its local phonon bath via a Holstein-type coupling. Light–matter interactions are treated using the Generalized Tavis–Cummings model, extending beyond the long-wavelength approximation. The total Hamiltonian, referred to as the *Generalized Holstein–Tavis–Cummings Hamiltonian* (GHTC) [1–3], is expressed as

$$\hat{H} = \hat{H}_{\text{ex}} + \hat{H}_{\text{ph}} + \hat{H}_{\text{ex-ph}} + \hat{H}_{\text{ex-b}} + \hat{H}_{\text{b}}, \tag{S1}$$

where $\hat{H}_{\text{ex}}$ denotes the excitonic Hamiltonian describing N non-interacting molecules, $\hat{H}_{\text{ph}}$ represents the photonic Hamiltonian of the cavity modes, $\hat{H}_{\text{ex-ph}}$ accounts for light–matter coupling, $\hat{H}_{\text{ex-b}}$ captures exciton–phonon interactions, and $\hat{H}_{\text{b}}$ describes the phonon bath associated with each molecule.

* spoddar3@ur.rochester.edu
† pengfei.huo@rochester.edu

The excitonic Hamiltonian is given by

$$\hat{H}_{\text{ex}} = (\hbar\omega_0 + \lambda) \sum_{n=0}^{N-1} \hat{\sigma}_n^\dagger \hat{\sigma}_n, \tag{S2}$$

where $\hat{\sigma}_n^\dagger$ and $\hat{\sigma}_n$ are the exciton creation and annihilation operators at site n, respectively, defined as

$$\hat{\sigma}_n^\dagger = |E_n, 0\rangle\langle G, 0|, \tag{S3a}$$
$$\hat{\sigma}_n = |G, 0\rangle\langle E_n, 0|. \tag{S3b}$$

The state $|E_n, 0\rangle$ represents an excitation localized at site n with no photons,

$$|E_n, 0\rangle = |e_n\rangle \bigotimes_{s\neq n} |g_s\rangle \bigotimes_k |0_k\rangle, \tag{S4}$$

while $|G, 0\rangle$ corresponds to the global ground state of the system,

$$|G, 0\rangle = \bigotimes_n |g_n\rangle \bigotimes_k |0_k\rangle. \tag{S5}$$

Here, the states $|g_n\rangle$ and $|e_n\rangle$ represent the ground and excited states at the n_{th} site, respectively and, $|0\rangle \equiv \bigotimes_k |0_k\rangle$ denotes the vacuum state of the electromagnetic field, with $|0_k\rangle$ representing the zero photon Fock state of the photonic mode with in-plane component of the wave vector k. The excitation energy between the electronic ground and excited states, identical for all N molecules, is $\hbar\omega_0 = E_e - E_g$, and λ represents the reorganization energy associated with exciton–phonon coupling. We consider a linear chain of molecules aligned along the x-axis, with the position of the n_{th} molecule given by $x_n = nL$, where L is the uniform intermolecular spacing. Periodic boundary conditions are applied such that $x_N \equiv x_0$.

For the photonic modes, the wave vector $\boldsymbol{k}$ comprises an in-plane component $k_\parallel$ (parallel to the BSW propagation direction, taken as the x-direction) and a perpendicular component $k_\perp$ (along the y-direction). In our one-dimensional system, only a single photonic mode is considered along the perpendicular direction, characterized by a fixed $k_\perp$ corresponding to the cavity energy $\hbar\omega_{\text{c}}$ at $k_\parallel = 0$. Since $k_\parallel$ is the only variable component of $\boldsymbol{k}$, we denote it simply as k, with the corresponding cavity mode frequency ω_k. The photonic Hamiltonian is then written as [2–4]

$$\hat{H}_{\text{ph}} = \sum_k \hbar\omega_k \hat{a}_k^\dagger \hat{a}_k, \tag{S6}$$

where $\hbar\omega_k$ is the energy of the photonic mode with the in-plane component of wave vector k, and $\hat{a}_k^\dagger$ ($\hat{a}_k$) are the corresponding photonic creation (annihilation) operators, defined as (with in the single photonic excitation subspace)

$$\hat{a}_k^\dagger = |G, 1_k\rangle\langle G, 0|, \tag{S7a}$$
$$\hat{a}_k = |G, 0\rangle\langle G, 1_k|. \tag{S7b}$$

Here, $|G, 1_k\rangle$ denotes the state with a single photon with the in-plane component of wave vector k and the excitonic ground state,

$$|G, 1_k\rangle = \bigotimes_n |g_n\rangle \otimes |1_k\rangle \bigotimes_{k'\neq k} |0_{k'}\rangle, \tag{S8}$$

where $|1_k\rangle$ represents the one-photon Fock state of the photonic mode with in-plane component of the wave vector k. We focus on a 1D BSW cavity characterized by a quasi-continuous in-plane direction (x). The BSW cavity dispersion relation is then given by

$$\hbar\omega_k = \hbar\omega_{\text{c}} + \left(\frac{\hbar c}{n_{\text{r}}}\right) |k|, \tag{S9}$$

where c is the speed of light, n_{r} is the refractive index of the cavity medium, and $|k|$ is the magnitude of the in-plane wavevector. The constant energy shift $\hbar\omega_{\text{c}}$ corresponds to the perpendicular (y) component of the confined mode and defines the cavity frequency at $k = 0$, i.e., $\hbar\omega_{k=0} = \hbar\omega_{\text{c}}$.

For a molecular chain of equally spaced emitters with inter-site separation L along the cavity surface, and under periodic boundary conditions [2], the in-plane wavevector is discretized as

$$k_\alpha = \frac{2\pi}{NL}\,\alpha, \tag{S10}$$

where α indexes the cavity modes. The total number of modes required to capture the relevant spectral range of the hybrid exciton–photon system is denoted by $\mathcal{M}$. The set of all k values associated with photon modes is denoted as $\{k_{\rm P}\}$, *i.e.*,

$$\{k_{\rm P}\} \equiv \{k_\alpha = \frac{2\pi}{NL}\alpha\}, \qquad \alpha \in \left[-\frac{\mathcal{M}-1}{2}, -\left(\frac{\mathcal{M}-1}{2}-1\right), \ldots, 0, \ldots, \left(\frac{\mathcal{M}-1}{2}-1\right), \frac{\mathcal{M}-1}{2}\right], \tag{S11}$$

when $\mathcal{M}$ is an odd number.

The light-matter interaction $\hat{H}_{\text{ex-ph}}$ Hamiltonian is expressed as [1–4]

$$\hat{H}_{\text{ex-ph}} = \hbar g_{\rm c} \sum_{k,n} (\hat{a}_k^\dagger \hat{\sigma}_n e^{-ikx_n} + \hat{\sigma}_n^\dagger \hat{a}_k e^{ikx_n}), \tag{S12}$$

where g_c denotes the single-molecule light–matter coupling strength, which is assumed to be independent of the photonic wave vector k.

Further, each exciton is coupled to its own independent, yet identical, phonon bath composed of local vibrational modes. The bath Hamiltonian is given by

$$\hat{H}_{\rm b} = \sum_{n=0}^{N-1} \sum_{v=1}^{N_v} \left(\frac{1}{2}\hat{p}_{n,v}^2 + \frac{1}{2}\omega_v^2 \hat{q}_{n,v}^2 \right), \tag{S13}$$

and the system–bath (exciton–phonon) interaction is described by

$$\hat{H}_{\text{ex-b}} = \sum_{n=0}^{N-1} \sum_{v=1}^{N_v} d_v\, \hat{\sigma}_n^\dagger \hat{\sigma}_n \left(\hat{b}_{n,v}^\dagger + \hat{b}_{n,v} \right) = \sum_{n=0}^{N-1} \sum_{v=1}^{N_v} \gamma_v\, \hat{\sigma}_n^\dagger \hat{\sigma}_n \hat{q}_{n,v}. \tag{S14}$$

Here, ω_v denotes the frequency of the $v_{\rm th}$ phonon mode, while $\hat{q}_{n,v}$ and $\hat{p}_{n,v}$ are the position and momentum operators, respectively, for the $v_{\rm th}$ vibrational mode associated with the $n_{\rm th}$ molecule. All phonon modes are assumed to have unit mass.

The phonon frequencies and coupling strength are sampled from the Debye spectral density [5, 6]:

$$\mathcal{J}(\omega) = \frac{\pi}{2} \sum_v \frac{\gamma_v^2}{\omega_v} \delta(\omega - \omega_v) = \frac{2\lambda \omega_{\rm b} \omega}{\omega_{\rm b}^2 + \omega^2}, \tag{S15}$$

where λ is the reorganization energy, as introduced in Eq. S2, and $\omega_{\rm b}$ is the characteristic frequency of the phonon bath. The reorganization energy λ can also be formally expressed as

$$\lambda = \frac{2}{\pi} \int_0^\infty d\omega\, \frac{\mathcal{J}(\omega)}{\omega} = \sum_v \frac{\gamma_v^2}{\omega_v^2}. \tag{S16}$$

For the Mean Field Ehrenfest (MFE) simulations, we sample Eq. S15 by using a total of $N_v = 20$ bath modes (for each molecule) with $\lambda = 36$ meV, and $\omega_b = 6$ meV. The bath parameters are sampled using the procedure outlined in Ref. 7.

B. Model Parameters

To model the exciton–photon system, we consider $N = 50001$ molecular sites arranged linearly with a uniform inter-site spacing of $L = 100$ Å. The system interacts with $\mathcal{M} = 4001$ photonic modes spanning the energy range $\hbar\omega_k \in [0.698, 3.898]$ eV. To reproduce the approximately linear dispersion of the bare Bloch surface wave (BSW) photonic modes and the exciton–polariton dispersions reported in Ref. 8, we adopt the following parameters: the excitonic transition energy $\hbar\omega_0 = 2.12$ eV, the cavity photon energy at normal incidence ($k_\parallel = 0$) $\hbar\omega_{\rm c} = 0.698$ eV, and a refractive index $n_{\rm r} = 1.54965$. The total light–matter coupling strength is set to $\sqrt{N}\, g_{\rm c} = 67$ meV, corresponding to a vacuum Rabi splitting of $\Omega_{\rm R} = 2\hbar\sqrt{N}\, g_{\rm c} = 134$ meV. The excitonic, photonic and the polaritonic dispersions are shown in figure S1.

The exciton–phonon interaction is modeled using a Drude–Lorentz spectral density characterized by a reorganization energy $\lambda = 36$ meV and a bath characteristic frequency $\hbar\omega_b = 6$ meV. These parameters collectively define the energetic and dynamical landscape used in all simulations.

II. BSW DISPERSION

For a planar Fabry-Pérot microcavity, the perpendicular wavevector component is quantized, $k_\perp = \frac{m\pi}{L_{\text{cav}}}$ with L_{cav} being the distance between the mirrors and $m \in [1, 2, 3, ...]$, and the photon dispersion is $\omega(k) = \frac{c}{n_r}\sqrt{k_\perp^2 + k^2}$, which is parabolic near $k = 0$. In contrast, a Bloch surface wave (BSW) is a surface-guided optical mode supported at the interface between a truncated dielectric multilayer (DBR) and a homogeneous medium. Its in-plane propagation is conveniently described by an effective index $n_{\text{BSW}}(\omega)$, defined through the phase-matching relation [9]

$$k = n_{\text{BSW}}(\omega)\,\frac{\omega}{c}, \tag{S17}$$

as commonly used in experimental characterizations of BSW propagation and dispersion. Over the relatively narrow spectral window relevant to our transport simulations, $n_{\text{BSW}}(\omega)$ varies weakly, so Eq. (S17) implies an approximately linear dispersion [8–11],

$$\omega(k) \approx \frac{c}{n_{\text{BSW}}}\,|k| \equiv \omega_c + \left(\frac{c}{n_r}\right)|k|, \tag{S18}$$

where the absolute value reflects left/right propagation symmetry, and the constant shift ω_c accounts for the chosen energy reference used to match the experimentally reported BSW branch in the plotted energy window.

III. POLARITONS AND DARK STATES

The polaritonic states are defined from the GHTC Hamiltonian by excluding the bath and exciton–phonon interaction terms. Accordingly, we consider the polaritonic Hamiltonian, $\hat{H}_{\text{pl}}$ [12] as

$$\hat{H}_{\text{pl}} = \hat{H}_{\text{ex}} + \hat{H}_{\text{ph}} + \hat{H}_{\text{ex-ph}}, \tag{S19}$$

which omits the exciton–phonon coupling term $\hat{H}_{\text{ex}-\text{b}}$ from the polaritonic Hamiltonian. This treatment contrasts with previous works that included exciton–phonon interactions within the polaritonic Hamiltonian itself [3, 13].

We define the collective excitonic bright ($|\text{B}_k\rangle$) and dark ($|\text{D}_{k'}\rangle$) states as

$$|\text{B}_k\rangle \equiv |E_k, 0\rangle = \frac{1}{\sqrt{N}}\sum_{n=0}^{N-1} e^{ikx_n}|E_n, 0\rangle, \quad k \in \{k_\text{P}\}, \tag{S20a}$$

$$|\text{D}_{k'}\rangle \equiv |E_{k'}, 0\rangle = \frac{1}{\sqrt{N}}\sum_{n=0}^{N-1} e^{ikx_n}|E_n, 0\rangle, \quad k' \in \{k_\text{D}\}, \tag{S20b}$$

where $\{k_\text{D}\}$ denotes the set of wave vectors that do not correspond to any photonic mode, defined as

$$\{k_\text{D}\} \equiv \{k_\eta = \frac{2\pi}{NL}\eta\} \tag{S21}$$

with,

$$\eta \in \left[-\frac{N-1}{2}, -\left(\frac{N-1}{2}-1\right), \ldots, -\left(\frac{\mathcal{M}-1}{2}+1\right), \left(\frac{\mathcal{M}-1}{2}+1\right), \ldots, \left(\frac{N-1}{2}-1\right), \frac{N-1}{2}\right], \tag{S22}$$

when N is an odd number. By construction, the two sets $\{k_\text{P}\}$ and $\{k_\text{D}\}$ are disjoint, i.e., $\{k_\text{P}\} \cap \{k_\text{D}\} = \emptyset$, and their union spans the entire (discretized) first Brillouin zone, $\{k_\text{P}\} \cup \{k_\text{D}\} = [-\pi/L, \ldots, \pi/L]$. The state $|E_k, 0\rangle$ denotes a collective exciton state with wave vector k and with no photons. From the cardinality of these sets, it follows that there are $\mathcal{M}$ bright excitonic states and $N - \mathcal{M}$ dark excitonic states. Unlike the bright states, the dark states do not participate in light–matter hybridization owing to the absence of photonic modes with matching in-plane wave vectors.

The polaritonic Hamiltonian, expressed in the reciprocal excitonic basis (in terms of the collective excitonic states), takes the form

$$\begin{aligned}\hat{H}_{\text{pl}} &= \hbar \sum_{k\in\{k_\text{P}\}} \left[\omega_0 \hat{\sigma}_k^\dagger \hat{\sigma}_k + \omega_k \hat{a}_k^\dagger \hat{a}_k + \sqrt{N} g_c \left(\hat{a}_k^\dagger \hat{\sigma}_k + \hat{\sigma}_k^\dagger \hat{a}_k\right)\right] + \hbar \sum_{k'\in\{k_\text{D}\}} \omega_0 \hat{\sigma}_{k'}^\dagger \hat{\sigma}_{k'}, \\ &= \hbar \sum_{k\in\{k_\text{P}\}} \left[\omega_0 |\text{B}_k\rangle\langle \text{B}_k| + \omega_k |G, 1_k\rangle\langle G, 1_k| + \sqrt{N} g_c \left(|G, 1_k\rangle\langle \text{B}_k| + |\text{B}_k\rangle\langle G, 1_k|\right)\right] + \hbar \sum_{k'\in\{k_\text{D}\}} \omega_0 |\text{D}_{k'}\rangle\langle \text{D}_{k'}|.\end{aligned} \tag{S23}$$

Here, $\hat{\sigma}_k^\dagger$ and $\hat{\sigma}_k$ are the creation and annihilation operators for a collective excitonic mode with wave vector k, defined as

$$\hat{\sigma}_k^\dagger = |E_k, 0\rangle\langle G, 0| = \hat{\sigma}_n^\dagger e^{ikx_n} \tag{S24a}$$

$$\hat{\sigma}_k = |G, 0\rangle\langle E_k, 0| = \hat{\sigma}_n e^{-ikx_n} \tag{S24b}$$

Due to light–matter coupling, the bright states $|\mathrm{B}_k\rangle$ hybridize with the corresponding photonic states $|G, 1_k\rangle$. In this model, there are $\mathcal{M}$ photonic states $\{|G, 1_k\rangle\}$ and $\mathcal{M}$ bright excitonic states (Eq. S20a). Their hybridization gives rise to $2\mathcal{M}$ polaritonic eigenstates—designated as upper and lower polariton branches—defined as [3, 14]

$$|+_k\rangle = \sin\phi_k\,|\mathrm{B}_k\rangle + \cos\phi_k\,|G, 1_k\rangle, \tag{S25a}$$

$$|-_k\rangle = \cos\phi_k\,|\mathrm{B}_k\rangle - \sin\phi_k\,|G, 1_k\rangle, \tag{S25b}$$

where $k \in \{k_\mathrm{P}\}$ and the mixing angle ϕ_k is given by [1, 14]

$$\phi_k = \frac{1}{2}\tan^{-1}\left(\frac{2\sqrt{N}g_\mathrm{c}}{\omega_k - \omega_0}\right), \qquad \phi_k \in \left[0, \frac{\pi}{2}\right]. \tag{S26}$$

The coefficients $\sin\phi_k$ and $\cos\phi_k$ are known as the Hopfield coefficients. The corresponding polariton energies are [3]

$$\omega_{\pm,k} = \frac{\omega_k + \omega_0}{2} \pm \frac{1}{2}\sqrt{(\omega_k - \omega_0)^2 + 4Ng_\mathrm{c}^2}. \tag{S27}$$

The polaritonic dispersion is shown in Figure S1 as color-coded lines, where the color indicates the excitonic character of each polaritonic state, *i.e.*, $\sin^2\phi_k$ and $\cos^2\phi_k$ for the upper and lower polariton branches, respectively.

In contrast, the dark excitonic states (Eq. S20b) remain decoupled from the photonic degrees of freedom, as no cavity modes exist with matching in-plane wave vectors. These diabatic definitions of polaritonic and dark states are used to analyze population dynamics in subsequent quantum dynamics simulations.

IV. DETAILS OF THE THEORETICAL SIMULATIONS

A. Preparation of initial quantum State

In several experimental studies of polaritonic transport [3, 8, 15], localized excitations with well-defined energy and momentum are typically generated using tightly focused pump pulses with narrow spectral bandwidth, often combined with momentum-selective probe schemes. Such excitation protocols create a localized nonequilibrium population of polaritons centered around a specific point on the lower polariton (LP) dispersion, enabling the subsequent dynamics to be resolved with both spatial and momentum precision. Inspired by these experimental approaches, the initial wavefunction in our simulations is constructed as a localized wavepacket composed of LP states lying close in energy to a chosen E_0.

To realize this, we first specify an energy value E_0 corresponding to the region of interest on the polaritonic dispersion for investigating the system dynamics. Ten values of E_0 are considered, ranging from 1.97 eV to 2.06 eV in equal increments of 0.01 eV. An energy width of $\Delta E = 15$ meV is selected, and the polaritonic states constituting the initial wavepacket are chosen according to the condition

$$\hbar\omega_{\pm,k} \in \left[E_0 - \tfrac{1}{2}\Delta E,\ E_0 + \tfrac{1}{2}\Delta E\right].$$

Since all selected E_0 values lie below the bare exciton energy, only the lower polariton (LP) states contribute to the construction of the initial wavefunction.

The set of k-values for which the energies of the LP states lie within the specified window is denoted by $\{k_\mathrm{w}\}$. Owing to the symmetry of the polariton dispersion relation about $k = 0$, this set contains pairs of equal and opposite wave vectors; that is, if $k_1 \in \{k_\mathrm{w}\}$, then $-k_1 \in \{k_\mathrm{w}\}$ as well. The initial wavefunction is then constructed as a normalized linear combination of these LP states, such that the resulting excitonic wavepacket in the site basis is approximately Gaussian, with its peak at the center of the simulation box, and has a width close to a prescribed value.

To achieve this, we first construct a Gaussian excitonic wavepacket in k-space, centered at the wave vector k_M within the set $\{k_\mathrm{w}\}$, such that $\hbar\omega_{\pm,k_\mathrm{M}}$ is the closest to E_0 value among $\{\hbar\omega_{\pm,k_\mathrm{w}}\}$.The excitonic wavepacket in reciprocal

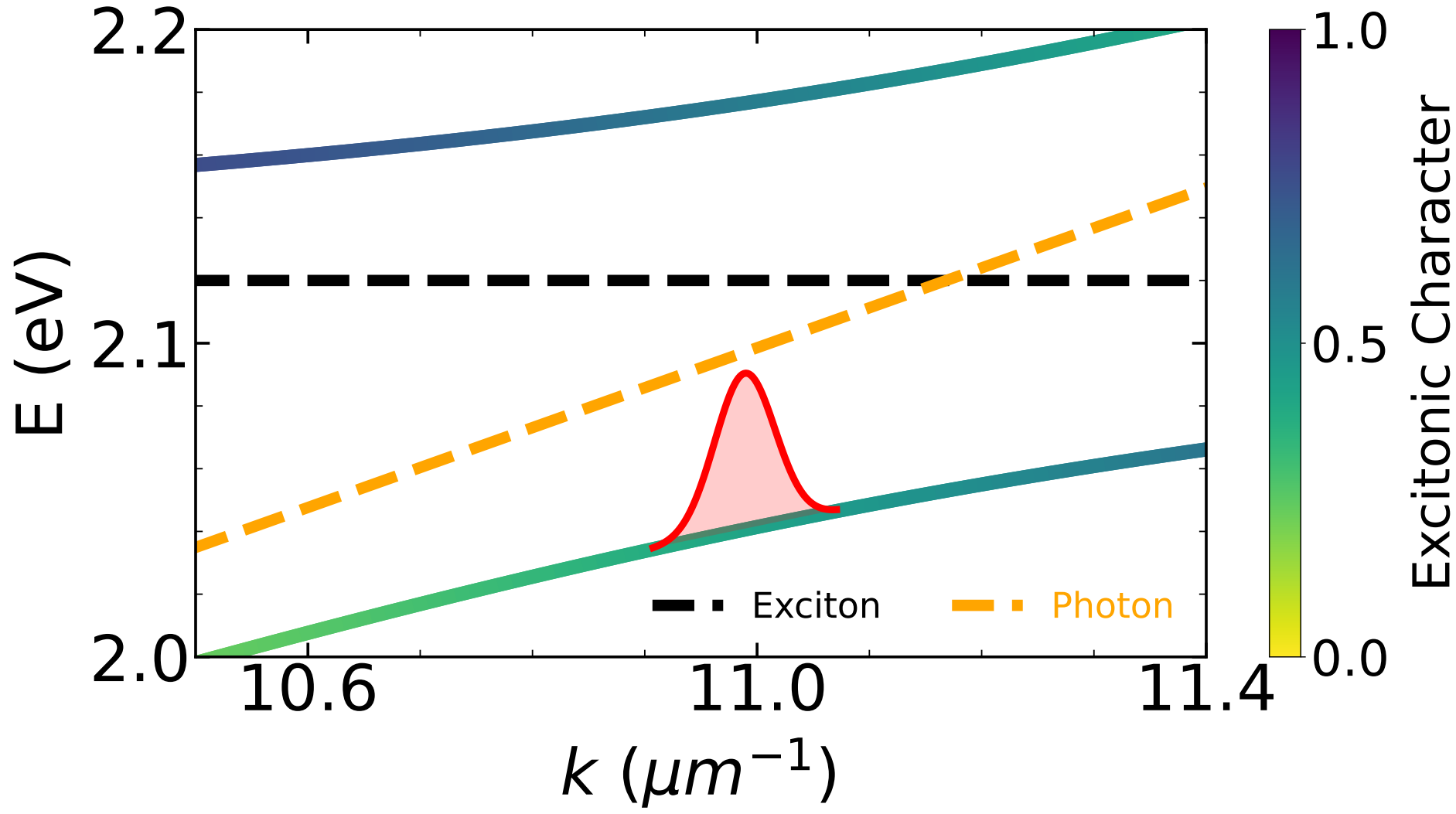

FIG. S1. Polariton dispersion relation showing the lower (LP) and upper (UP) polariton branches. The excitonic (black dashed) and photonic (orange dashed) dispersions are plotted for reference. The color scale indicates the Excitonic character ($|\alpha_{\rm ex}|^2$) of the polaritonic states, ranging from photonic (yellow) to excitonic (dark violet). The Gaussian wavepacket on the LP branch schematically shows the location of the initial wavepacket in k-space for $E_0 = 2.04$ eV.

space is taken as a superposition of two Gaussian functions, one centered at $k_{\rm M}$ and the other at $-k_{\rm M}$. Explicitly, we define

$$\bar{c}_{k_{\rm w}} = \left[e^{-\frac{(k_{\rm w}-k_{\rm M})^2}{2}\Delta x^2} + e^{-\frac{(k_{\rm w}+k_{\rm M})^2}{2}\Delta x^2} \right] e^{-\frac{ik_{\rm w}NL}{2}}, \tag{S28}$$

where $\{\bar{c}_{k_{\rm w}}\}$s are initial coefficients of the reciprocal excitonic states $\{|E_{k_{\rm w}}, 0\rangle\}$. Here, Δx controls the spatial width of the wavepacket, and its value is kept fixed across different choices of E_0 so that the initial excitonic wavepacket has nearly the same width for all initial conditions. The phase factor $e^{-\frac{ik_{\rm w}NL}{2}}$ ensures that the wavepacket is centered at $x = (N/2)L$.

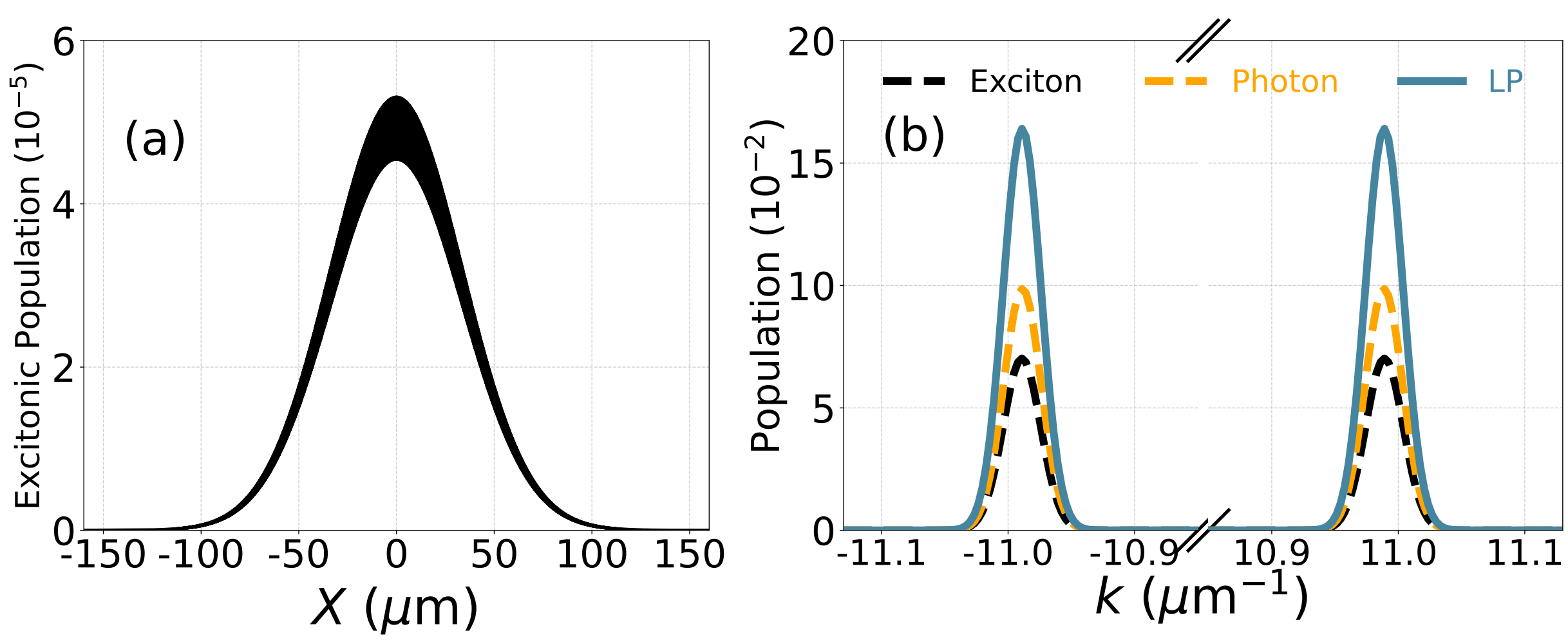

FIG. S2. Population of the initial state for $E_0 = 2.04$ eV. (a) Initial excitonic population distribution in the site basis. While computing the site positions, the site index grid was shifted from $-\frac{N}{2}$ to $\frac{N}{2}$ such that the center of the simulation box lies at the origin. (b) Initial excitonic, photonic, and lower polaritonic (LP) populations in reciprocal k-space.

The amplitude $c_{k_{\rm w}}$ of the photonic state $\{|G, 1_{k_{\rm w}}\rangle\}$ is calculated using the relative proportion of the exciton-photon in a particular LP state at $k = k_{\rm w}$, which is controlled by the mixing angle $\phi_{k_{\rm w}}$.

$$c_{k_{\rm w}} = -\bar{c}_{k_{\rm w}} \tan \phi_{k_{\rm w}}. \tag{S29}$$

The corresponding site-basis excitonic coefficients are then obtained by taking a discrete inverse Fourier transform of the reciprocal basis excitonic coefficients as

$$c_n = \frac{1}{\sqrt{N}} \sum_{k \in k_\mathrm{w}} \bar{c}_k e^{iknL} = \frac{1}{\sqrt{N}} \sum_{k \in k_\mathrm{w}} \left[e^{-\frac{(k-k_\mathrm{M})^2}{2}\Delta x^2} + e^{-\frac{(k+k_\mathrm{M})^2}{2}\Delta x^2} \right] e^{-ik(n_0-n)L}. \tag{S30}$$

So finally, our initial state for any E_0 value would look like

$$\begin{aligned} |\psi(0)\rangle &= \sum_{n=0}^{N-1} c_n |E_n, 0\rangle + \sum_{k \in \{k_\mathrm{w}\}} c_k |G, 1_k\rangle, \\ &= \sum_{k \in k_\mathrm{w}} \left[e^{-\frac{(k-k_\mathrm{M})^2}{2}\Delta x^2} + e^{-\frac{(k+k_\mathrm{M})^2}{2}\Delta x^2} \right] e^{-ikn_0L} \left(\frac{1}{\sqrt{N}} \sum_{n=0}^{N-1} e^{ikn} |E_n, 0\rangle - \tan\phi_k |G, 1_k\rangle \right), \end{aligned} \tag{S31}$$

where the wavevector window $\{k_\mathrm{w}\}$ and central momentum k_M are determined by the chosen energy E_0. The total wavefunction is normalized prior to initiating the dynamics.

Assuming that the k values are nearly continuous, one can approximate the summation by an integral:

$$c_n = \frac{1}{\sqrt{N}} \int_{k \in k_\mathrm{w}} \left[e^{-\frac{(k-k_\mathrm{M})^2}{2}\Delta x^2} + e^{-\frac{(k+k_\mathrm{M})^2}{2}\Delta x^2} \right] e^{-ik(n_0-n)L} dk. \tag{S32}$$

Since Δx is chosen such that the Gaussian function decays rapidly beyond a certain k-value, the integration limits can be extended to $\pm\infty$:

$$c_n = \frac{1}{\sqrt{N}} \int_{-\infty}^{\infty} \left[e^{-\frac{(k-k_\mathrm{M})^2}{2}\Delta x^2} + e^{-\frac{(k+k_\mathrm{M})^2}{2}\Delta x^2} \right] e^{-ik(n_0-n)L} dk. \tag{S33}$$

Using standard Gaussian integral techniques, it can be shown that c_n is also a Gaussian function, centered at $x = n_0L$ with a width proportional to Δx. However, since the k-values are discrete rather than truly continuous, minor fluctuations appear in c_n, though it retains an overall Gaussian profile.

This preparation effectively mimics the experimental scenario in which a focused pump pulse excites a small subset of LP states near a particular energy and momentum, enabling us to investigate the subsequent transport dynamics under realistic excitation conditions. The initial wave packet corresponding to $E_0 = 2.04$ eV is schematically illustrated in the dispersion plot in Figure S1, and the associated initial population is presented in Figure S2.

B. Sampling initial Phonon positions and momenta

Initial bath conditions sampled from the analytic form of the Wigner distribution for a Gaussian bath

$$[\hat{\rho}_\mathrm{R}]_\mathrm{W}(\mathbf{R}, \mathbf{P}) = \prod_{n,v} 2\tanh\left(\frac{\hbar\omega_v}{2k_\mathrm{B}T}\right) \exp\left\{ -\tanh\left(\frac{\hbar\omega_v}{2k_\mathrm{B}T}\right) \left(\frac{\omega_v R_{n,v}^2}{\hbar} + \frac{P_{n,v}^2}{\hbar\omega_v} \right) \right\} \tag{S34}$$

which are sampled using the Gaussian random number generator based on the Box–Muller method.

C. Ehrenfest Propagation Scheme Using Chebyshev Algorithm

In this work, all simulations were performed in the site basis for excitons and in the k-basis for photons. We employed mean-field Ehrenfest dynamics to propagate the coupled system, wherein the exciton–photon degrees of freedom (DOF) are treated quantum mechanically, while the phonon bath is treated classically.

The time evolution of the quantum subsystem is governed by the time-dependent Schrödinger equation (TDSE):

$$i\hbar \frac{\partial}{\partial t} |\psi(t)\rangle = \hat{H}_\mathrm{Q}(\mathbf{q}(t)) |\psi(t)\rangle, \tag{S35}$$

where $\hat{H}_{\rm Q} = \hat{H} - \hat{H}_{\rm b}$ represents the quantum part of the Hamiltonian, which includes the excitonic and photonic DOF, as well as the exciton–phonon coupling. The phonon coordinates and momenta are evolved according to the mean force exerted on them by the quantum subsystem.

The phonon positions and momenta were updated every 1 fs. Between two consecutive updates of the phonon variables, the wavefunction was propagated twice using a quantum time step of $dt_{\rm Q} = 0.5$ fs. A schematic breakdown of one complete propagation cycle is outlined below.

At a given time t, the system is described by the wavefunction $|\psi(t)\rangle$ and the phonon positions and momenta $\{q_{n,v}(t)\}$ and $\{p_{n,v}(t)\}$. One full Ehrenfest update proceeds as follows:

1. **Quantum update:** The wavefunction is propagated for one quantum step of $dt_{\rm Q} = 0.5$ fs according to

$$i\hbar \frac{d}{dt}|\psi(t)\rangle = \hat{H}_{\rm Q}(t)|\psi(t)\rangle. \tag{S36}$$

2. **Classical (phonon) update:** Using the updated $|\psi(t)\rangle$, the phonon positions and momenta are advanced by $dt_{\rm C} = 1$ fs via

$$\frac{\partial q_{n,v}(t)}{\partial t} = \big\langle \psi(t) \big| \frac{\partial \hat{H}}{\partial p_{n,v}(t)} \big| \psi(t) \big\rangle = \frac{p_{n,v}(t)}{m}, \tag{S37}$$

$$\begin{aligned}\frac{\partial p_{n,v}(t)}{\partial t} &= -\big\langle \psi(t) \big| \frac{\partial \hat{H}}{\partial q_{n,v}(t)} \big| \psi(t) \big\rangle \\ &= -m\omega_v^2 q_{n,v}(t) - \gamma_v \big\langle \psi(t) \big| \hat{\sigma}_n^\dagger \hat{\sigma}_n \big| \psi(t) \big\rangle.\end{aligned} \tag{S38}$$

3. **Quantum update (with updated bath):** The exciton–phonon coupling Hamiltonian $\hat{H}_{\rm ex-b}$ is recalculated using the updated phonon coordinates, and the wavefunction is again propagated for another $dt_{\rm Q} = 0.5$ fs according to Eq. S36.

The quantum propagation in Eq. S36 was performed using the Chebyshev algorithm, while the classical phonon dynamics were evolved using the velocity-Verlet algorithm. The relatively large quantum time step was feasible due to the significantly lower numerical error of the Chebyshev propagator compared to the Verlet or fourth-order Runge–Kutta (RK4) methods at the same step size. All results are obtained with an ensemble of 240 independent trajectories.

The Chebyshev Propagation Algorithm

To advance the wavefunction for a short time step δt, we can use

$$\begin{aligned}|\psi(t+\delta t)\rangle &= \hat{U}(t+\delta t, t)|\psi(t)\rangle, \\ &= e^{-\frac{i}{\hbar}\hat{H}_{\rm Q}(t)\delta t}|\psi(t)\rangle,\end{aligned} \tag{S39}$$

where $\hat{U}(t+\delta t, t)$ is the time-evolution operator. To evaluate $\hat{U}(t+\delta t, t)|\psi(t)\rangle$, we employ a Chebyshev expansion [16, 17]:

$$\hat{U}(t+\delta t, t)|\psi(t)\rangle = b_0(z)\,|\psi^{(0)}(t)\rangle + \sum_{l=1}^{\infty} \phi_l(z)\, b_l(z)\, |\psi^{(l)}(t)\rangle, \tag{S40}$$

where

$$|\psi^{(l)}(t)\rangle = \begin{cases} |\psi(t)\rangle, & l = 0, \\ \hat{\mathcal{H}}(t)|\psi(t)\rangle, & l = 1, \\ 2\hat{\mathcal{H}}(t)|\psi^{(l-1)}(t)\rangle - |\psi^{(l-2)}(t)\rangle, & l \geq 2, \end{cases} \tag{S41}$$

and

$$\hat{\mathcal{H}}(t) = \frac{2}{\delta_E}\left[\hat{H}_{\rm Q}(t) - \left(\epsilon_{\rm min} + \tfrac{\delta_E}{2}\right)\hat{\mathcal{I}}\right], \tag{S42}$$

with $\hat{\mathcal{I}}$ being the identity operator. Here, $\epsilon_{\rm min}$ denotes the lowest eigenvalue of $\hat{H}_{\rm Q}$, and δ_E represents the spectral radius of the system, *i.e.*, the difference between the largest and smallest eigenvalues of $\hat{H}_{\rm Q}$. In the Chebyshev scheme,

approximate estimates of these two quantities are sufficient. We use $\epsilon_{\text{min}} \approx \hbar\omega_{-,k=0} - \lambda$ and $\delta_E \approx \hbar\omega_{+,k=\frac{\mathcal{M}\pi}{NL}} - \hbar\omega_{-,k=0} + 2\lambda$. The midpoint term, $(\epsilon_{\text{min}} + \frac{\delta_E}{2})$, can thus be viewed as the average energy eigenvalue ϵ_{avg}.

The coefficients $\{b_l(z)\}$ are Bessel functions of the first kind with $z = \frac{\delta t\,\delta_E}{2\hbar}$, and the phase factor $\phi_l(z)$ is defined as

$$\phi_l(z) = 2i^l \exp\left(-\frac{i}{\hbar}\left[\frac{\delta_E}{2} + \epsilon_{\text{min}}\right]\delta t\right) = 2i^l \exp\left(-\frac{i}{\hbar}\epsilon_{\text{avg}}\delta t\right). \tag{S43}$$

Because $\phi_l(z)$ depends on both ϵ_{min} and δt, the accuracy of the propagation may degrade when $|\epsilon_{\text{min}}|$ is large, as rapid oscillations in $\phi_l(z)$ can lead to accumulated numerical noise over time. A similar challenge also arises in other propagation schemes such as RK4.

To mitigate this numerical instability, we shift the overall energy reference by subtracting the mean energy from the diagonal elements of $\hat{H}_{\text{Q}}(t)$. Specifically, the Hamiltonian is redefined as $\hat{H}'_{\text{Q}}(t) = \hat{H}_{\text{Q}}(t) - \epsilon_{\text{avg}}\hat{\mathcal{I}}$, so that the new energy minimum becomes $\epsilon'_{\text{min}} = -\frac{\delta_E}{2}$ and the average energy ϵ_{avg} is approximately zero. This renders the phase factor time-independent, $\phi'_l(z) = 2i^l$ [16, 17].

Equation S39 can then be rewritten as

$$|\psi(t+\delta t)\rangle = e^{-\frac{i}{\hbar}\hat{H}_{\text{Q}}(t)\delta t}|\psi(t)\rangle, \tag{S44}$$

$$= e^{-\frac{i}{\hbar}\epsilon_{\text{avg}}\hat{\mathcal{I}}\delta t}\, e^{-\frac{i}{\hbar}\hat{H}'_{\text{Q}}(t)\delta t}|\psi(t)\rangle, \tag{S45}$$

$$= e^{-\frac{i}{\hbar}\epsilon_{\text{avg}}\hat{\mathcal{I}}\delta t}\, \hat{U}'(t+\delta t, t)|\psi(t)\rangle.$$

The first exponential term, being a constant global phase, can be applied analytically. For the remaining term $\hat{U}'(t+\delta t, t)|\psi(t)\rangle$, we use an expression analogous to Eq. S40, but with the shifted quantities $\phi'_l(z)$ and $\hat{\mathcal{H}}'(t) = \frac{2}{\delta_E}\hat{H}'_{\text{Q}}(t)$, following the same recursive structure as Eq. IV C. The series in Eq. S40 is truncated after a finite number of terms. In our implementation, the number of terms (l_{cheb}) retained depends on the value of z, and the truncation is determined by a cutoff value of the Bessel function coefficint. To be more specific we chose l_{cheb} such that $b_{l_{\text{cheb}}}(z) > 10^{-10}$ while $b_{l_{\text{cheb}}+1}(z) < 10^{-10}$. According to this threshold, a total of 12 terms are included in the computation.

Efficient Hamiltonian action on Wavefunction

For the evaluation of $\hat{\mathcal{H}}(t)|\psi(t)\rangle$, we note that

$$\hat{\mathcal{H}}(t)|\psi(t)\rangle = \frac{2}{\delta_E}\hat{H}_{\text{Q}}(t)|\psi(t)\rangle - \left(2\frac{\epsilon_{\text{min}}}{\delta_E} + 1\right)\hat{\mathcal{I}}|\psi(t)\rangle. \tag{S46}$$

As evident, the most computationally demanding term in Eq. S46 is the matrix–vector multiplication $\hat{H}_{\text{Q}}(t)|\psi(t)\rangle$, which scales as $\mathcal{O}(\mathcal{N}^2)$ if evaluated directly. The remaining terms involve scalar–vector operations that scale linearly as $\mathcal{O}(\mathcal{N})$, where $\mathcal{N} = N + \mathcal{M}$ is the total number of quantum degrees of freedom.

The computation of $\hat{H}_{\text{Q}}(t)|\psi(t)\rangle$ can be significantly optimized by exploiting the sparsity and block symmetry of $\hat{H}_{\text{Q}}(t)$ [12]. Writing the total wavefunction as a direct sum,

$$|\psi(t)\rangle = |\psi_{\text{ex}}(t)\rangle \oplus |\psi_{\text{ph}}(t)\rangle,$$

the action of the Hamiltonian on the state vector becomes

$$\begin{aligned}
\hat{H}_{\text{Q}}(t)|\psi(t)\rangle &= \left[\sum_{n=0}^{N-1}\left(\hbar\omega_0 + \lambda + \sum_{v=1}^{N_v}\gamma_v\, q_{n,v}\right)\hat{\sigma}_n^\dagger\hat{\sigma}_n + \sum_{k\in\{k_{\text{P}}\}}\hbar\omega_k\hat{a}_k^\dagger\hat{a}_k + \hbar g_{\text{c}}\sum_{k,n}\left(\hat{a}_k^\dagger\hat{\sigma}_n e^{-ikx_n} + \hat{\sigma}_n^\dagger\hat{a}_k e^{ikx_n}\right)\right]|\psi(t)\rangle \\
&= \left[\hat{\mathcal{D}}_{\text{ex}}|\psi_{\text{ex}}(t)\rangle \oplus \hat{\mathcal{D}}_{\text{ph}}|\psi_{\text{ph}}(t)\rangle\right] + \left[\sqrt{N}\hbar g_{\text{c}}\sum_{k\in\{k_{\text{P}}\}}(\hat{a}_k^\dagger\sigma_k + \hat{\sigma}_k^\dagger\hat{a}_k)|\psi(t)\rangle\right] \\
&\equiv \left[\hat{\mathcal{D}}_{\text{ex}}|\psi_{\text{ex}}(t)\rangle \oplus \hat{\mathcal{D}}_{\text{ph}}|\psi_{\text{ph}}(t)\rangle\right] + \left[\hat{\mathcal{P}}_{\text{ex-ph}}|\psi(t)\rangle\right].
\end{aligned} \tag{S47}$$

Here, $\hat{\mathcal{D}}_{\text{ex}}$ and $\hat{\mathcal{D}}_{\text{ph}}$ are diagonal matrices in the excitonic and photonic subspaces, respectively, with diagonal entries $\{\hbar\omega_0 + \lambda + \sum_{v=1}^{N_v}\gamma_v q_{n,v}\}$ and $\{\hbar\omega_k\}$ respectively. Their action on the corresponding wavefunction components reduces to scalar–vector multiplication, scaling as $\mathcal{O}(N)$.

The coupling matrix $\hat{\mathcal{P}}_{\text{ex-ph}}$ possesses a symmetric and sparse block structure when expressed in the excitonic k-basis (the photonic component $|\psi_{\text{ph}}(t)\rangle$ is already represented in k-space in our simulations). Its schematic form is

$$\hat{\mathcal{P}}_{\text{ex-ph}} = \left[\begin{array}{cc} \hat{O}_{\text{ex}} & \begin{array}{ccccc} 0 & 0 & 0 & \cdots & 0 \\ 0 & 0 & 0 & \cdots & 0 \\ \vdots & \vdots & \vdots & \ddots & \vdots \\ \sqrt{N}g_c & 0 & 0 & \cdots & 0 \\ 0 & \sqrt{N}g_c & 0 & \cdots & 0 \\ \vdots & \vdots & \ddots & \cdots & 0 \\ 0 & 0 & 0 & \cdots & \sqrt{N}g_c \\ \vdots & \vdots & \vdots & \cdots & \vdots \\ 0 & 0 & 0 & \cdots & 0 \end{array} \\ \begin{array}{ccccccccc} 0 & 0 & \cdots & \sqrt{N}g_c & 0 & \cdots & 0 & \cdots & 0 \\ 0 & 0 & \cdots & 0 & \sqrt{N}g_c & \cdots & 0 & \cdots & 0 \\ 0 & 0 & \cdots & 0 & 0 & \cdots & 0 & \cdots & 0 \\ \vdots & \vdots & \ddots & \vdots & \ddots & \ddots & \vdots & \cdots & 0 \\ 0 & 0 & 0 & \cdots & 0 & \cdots & \sqrt{N}g_c & \cdots & 0 \end{array} & \hat{O}_{\text{ph}} \end{array}\right].$$

where $\hat{O}_{\text{ex}}$ and $\hat{O}_{\text{ph}}$ denote zero matrices in the excitonic and photonic subspaces respectively.

Since the excitonic part of the wavefunction is represented in the site basis in our simulations, we exploit the symmetry of $\hat{\mathcal{P}}_{\text{ex-ph}}$ by transforming $|\psi_{\text{ex}}(t)\rangle$ to the momentum (k) basis via a Fourier transform. In this representation, the multiplication $\hat{\mathcal{P}}_{\text{ex-ph}}|\psi(t)\rangle$ reduces to simple scalar–vector operations. After this step, an inverse Fourier transform is applied to the excitonic part of the product vector to return to the site basis, and the resulting vector is added to the diagonal contributions $\left[\hat{\mathcal{D}}_{\text{ex}}|\psi_{\text{ex}}(t)\rangle \oplus \hat{\mathcal{D}}_{\text{ph}}|\psi_{\text{ph}}(t)\rangle\right]$.

This algorithm dramatically reduces computational cost when the Fourier transforms are implemented using the Fast Fourier Transform (FFT) algorithm, which scales as $\mathcal{O}(\mathcal{N}\log\mathcal{N})$. Consequently, all other operations—scalar–vector multiplications and vector additions—become negligible compared to the FFT cost, yielding an overall scaling of $\mathcal{O}(\mathcal{N}\log\mathcal{N})$, a substantial improvement over the brute-force $\mathcal{O}(\mathcal{N}^2)$ scaling, particularly for the large systems simulated here ($\mathcal{N} \sim 10^5$). A detailed computational analysis of this optimization, including its extension to k-dependent exciton–photon coupling g_{c}, can be found in Ref. 12.

V. CALCULATION OF TRANSPORT PROPERTIES

A. Spatial Distribution of the Polariton Wavepacket

At any time t, the state of the system in our simulation can be expressed as

$$|\psi(t)\rangle = \sum_{n=0}^{N-1} c_n(t)|E_n\rangle + \sum_{\alpha} c_k(t)|k\rangle \equiv |\psi_{\text{ex}}(t)\rangle + |\psi_{\text{ph}}(t)\rangle, \tag{S48}$$

To interpret the spatial distribution of the polariton wavepacket at time t and position x_n, we define the polariton states in real space $|\pm_n\rangle$, by taking a discrete Fourier transform of the polariton states in k-basis (Eq. 7). This leads

to the following expressions

$$
\begin{aligned}
|+_n\rangle &= \frac{1}{\sqrt{N}}\sum_{k\in\{k_\mathrm{P}\}} e^{-ikx_n}|+_k\rangle = \frac{1}{\sqrt{N}}\sum_{k\in\{k_\mathrm{P}\}} e^{-ikx_n}\left[\sin\phi_k|E_k,0\rangle + \cos\phi_k|G,1_k\rangle\right], \\
&= \sum_{k\in\{k_\mathrm{P}\}}\left[\frac{\sin\phi_k}{N}\sum_{n'=0}^{N-1} e^{-ik(x_n-x_{n'})}|E_{n'},0\rangle + \frac{\cos\phi_k}{\sqrt{N}}e^{-ikx_n}|G,1_k\rangle\right],
\end{aligned}
\tag{S49a}
$$

$$
\begin{aligned}
|-_n\rangle &= \frac{1}{\sqrt{N}}\sum_{k\in\{k_\mathrm{P}\}} e^{-ikx_n}|-_k\rangle = \frac{1}{\sqrt{N}}\sum_{k\in\{k_\mathrm{P}\}} e^{-ikx_n}\left[\cos\phi_k|E_k,0\rangle - \sin\phi_k|G,1_k\rangle\right], \\
&= \sum_{k\in\{k_\mathrm{P}\}}\left[\frac{\cos\phi_k}{N}\sum_{n'=0}^{N-1} e^{-ik(x_n-x_{n'})}|E_{n'},0\rangle - \frac{\sin\phi_k}{\sqrt{N}}e^{-ikx_n}|G,1_k\rangle\right].
\end{aligned}
\tag{S49b}
$$

The spatial distribution of the polariton wavepacket is thus given by $|\psi_\pm(x_n,t)|^2 = |\langle\pm_n|\psi(t)\rangle|^2$, and the full expression for $|\psi_\pm(x_n,t)|^2$ is

$$
\begin{aligned}
|\psi_+(x_n,t)|^2 = &\left|\sum_{k\in\{k_\mathrm{P}\}}\sum_{n'=0}^{N-1}\frac{\sin\phi_k}{N}e^{-ik(x_n-x_{n'})}c_{n'}\right|^2 + \left|\sum_{k\in\{k_\mathrm{P}\}}\frac{\cos\phi_k}{\sqrt{N}}e^{-ikx_n}c_k\right|^2 \\
&+ 2\mathrm{Re}\left[\sum_{k\in\{k_\mathrm{P}\}}\sum_{n'=0}^{N-1}\frac{\sin\phi_k}{N}e^{-ik(x_n-x_{n'})}c_{n'} \times \left(\sum_{k'\in\{k_\mathrm{P}\}}\frac{\cos\phi_{k'}}{\sqrt{N}}e^{-ik'x_n}c_{k'}\right)^*\right],
\end{aligned}
\tag{S50a}
$$

$$
\begin{aligned}
|\psi_-(x_n,t)|^2 = &\left|\sum_{k\in\{k_\mathrm{P}\}}\sum_{n'=0}^{N-1}\frac{\cos\phi_k}{N}e^{-ik(x_n-x_{n'})}c_{n'}\right|^2 + \left|\sum_{k\in\{k_\mathrm{P}\}}\frac{\sin\phi_k}{\sqrt{N}}e^{-ikx_n}c_k\right|^2 \\
&- 2\mathrm{Re}\left[\sum_{k\in\{k_\mathrm{P}\}}\sum_{n'=0}^{N-1}\frac{\cos\phi_k}{N}e^{-ik(x_n-x_{n'})}c_{n'} \times \left(\sum_{k'\in\{k_\mathrm{P}\}}\frac{\sin\phi_{k'}}{\sqrt{N}}e^{-ik'x_n}c_{k'}\right)^*\right].
\end{aligned}
\tag{S50b}
$$

The last term in Eqs. S50a and S50b describe the interference between the excitonic part and the photonic part of the polariton wavepacket. These *interference* terms between the exciton wavepacket and photonic wavepacket, which were included in the Gross–Pitaevskii equation [18], have a non-negligible contribution in computing polariton MSDs and group velocities, see Ref. 12 for details.

Likewise, the dark state wavepacket in real space is obtained as

$$
|\mathrm{D}_n\rangle = \frac{1}{N}\sum_{k'\in\{k_D\}}\sum_{n'=0}^{N-1} e^{-ik'(x_n-x_{n'})}|E_{n',0}\rangle. \tag{S51}
$$

The spatial distribution of the dark state wavepacket is given by $|\psi_\mathrm{D}(x_n,t)|^2 = |\langle\mathrm{D}_n|\psi(t)\rangle|^2$, and is computed as

$$
|\psi_\mathrm{D}(x_n,t)|^2 = \left|\frac{1}{N}\sum_{k'\in\{k_\mathrm{D}\}}\sum_{n'=0}^{N-1} e^{-ik'(x_n-x_{n'})}c_{n'}\right|^2 \tag{S52}
$$

We compute the polariton group velocities and MSDs from the population contributions at n_th site coming from the polariton and dark state wavepackets, that is

$$
P_n = |\psi(x_n,t)|^2 = |\psi_\pm(x_n,t)|^2 + |\psi_\mathrm{D}(x_n,t)|^2. \tag{S53}
$$

B. Calculations of Group Velocity

After performing the dynamics, we tracked the tail of the polaritonic wavepacket in the site basis to extract the group velocity. The procedure follows approaches employed in previous studies of polaritonic transport [3, 15]. If $P_n(t)$ denotes the polaritonic population (LP+Dark) on the n_th site at time t, the position of the wavepacket tail

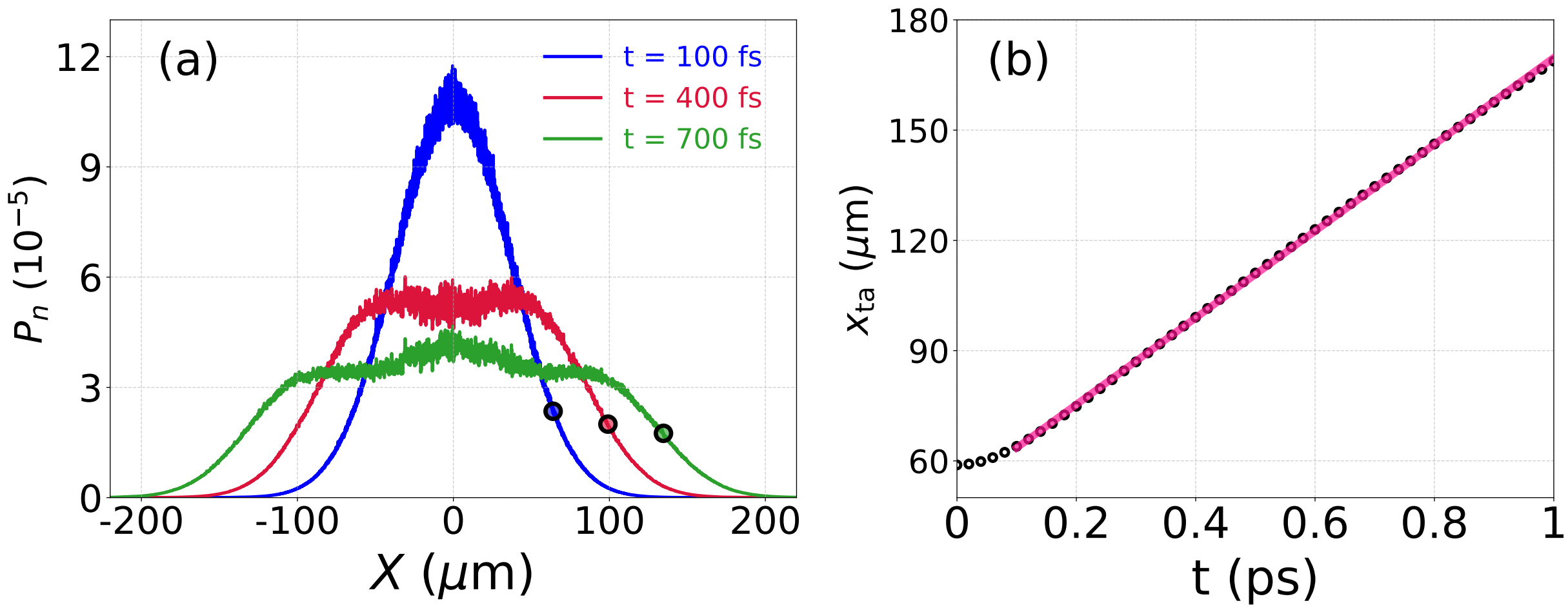

FIG. S3. Extraction of the group velocity by tracking the tail of the wave packet. (a) Population of LP+Dark states in the site basis at three different times during the dynamics ($t = 100,\ 400,\ 700$ fs) for $E_0 = 2.00$ eV. While computing the site positions, the site index grid was shifted from $-\frac{N}{2}$ to $\frac{N}{2}$ such that the center of the simulation box lies at the origin. The black open circles indicate the instantaneous position of the wave-packet tail at those times. (b) The tail positions (black open circles) are plotted as a function of time. The pink line is a linear fit, whose slope yields the group velocity, $\tilde{v}_{g,-}$.

at a given time was identified as the site where the cumulative population first exceeds 4% of the total polaritonic population, *i.e.*,

$$\sum_{n=0}^{m-1} P_n(t) < 0.04 P_{\rm pol}(t) \quad \text{and} \quad \sum_{n=0}^{m} P_n(t) \geq 0.04 P_{\rm pol}(t), \tag{S54}$$

such that

$$x_{\rm ta} = mL, \tag{S55}$$

where $P_{\rm pol}(t) = \sum_{n=0}^{N-1} P_n(t)$ is the total polaritonic population at time t. The group velocity was then defined as the absolute rate of change of the tail position of the polaritonic wavepacket,

$$\tilde{v}_{g,-} = \left| \frac{dx_{\rm ta}}{dt} \right|. \tag{S56}$$

The polaritonic wave packet at different times, along with the extracted tail positions for $E_0 = 2.00$ eV, is shown in Fig. S3a. Figure S3b displays the corresponding tail locations as a function of time, together with a linear fit whose slope yields the group velocity.

C. Calculation of MSD

At any time step, the normalized mean square displacement (MSD) of the polaritonic wavepacket was computed using the standard definition,

$$\sigma_t^2 = \langle X^2(t) \rangle - \langle X(t) \rangle^2 = \frac{1}{\sum_n P_n(t)} \sum_n x_n^2 P_n(t) - \left(\frac{1}{\sum_n P_n(t)} \sum_n x_n P_n(t) \right)^2. \tag{S57}$$

D. Extraction of the diffusion constant

The temporal evolution of the mean–squared displacement, MSD(t), was first quantified and fitted to a power–law dependence, MSD(t) $\propto t^{\beta}$, in different time windows to extract the transport exponent β. This analysis allows us to distinguish between ballistic ($\beta \approx 2$) and diffusive ($\beta \approx 1$) regimes. For those initial conditions (i.e., values of E_0)

that exhibit a region where $\beta \approx 1$, the dynamics in that interval is diffusive. In that time window, the MSD was further fitted linearly in time, and the extracted slope was set equal to $2\mathcal{D}$, *i.e.*

$$\mathrm{MSD}(t) = 2\mathcal{D}\, t, \tag{S58}$$

allowing us to determine the diffusion coefficient $\mathcal{D}$.

As an example, Fig. S4 shows the full evolution of $\mathrm{MSD}(t)$ for $E_0 = 2.02$ eV, together with the corresponding linear fit in the $\beta \approx 1$ region. The quantity $\mathcal{D}$ is obtained as one–half of the fitted slope. This figure explicitly illustrates the fitting procedure applied throughout the manuscript.

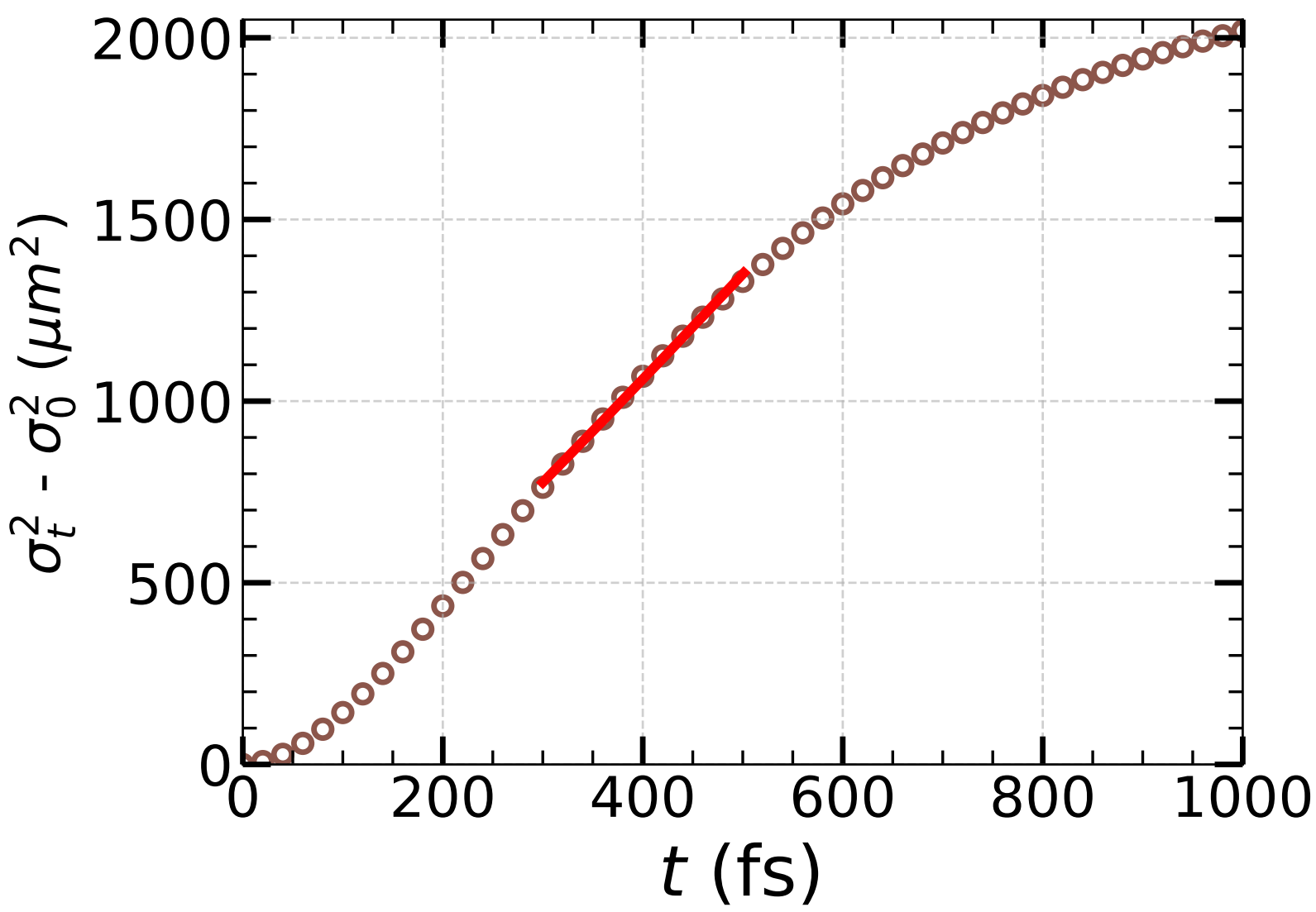

FIG. S4. Extraction of the diffusion coefficient $\mathcal{D}$ by linearly fitting $\mathrm{MSD}(t)$ with time in the region where $\beta \approx 1$ for $E_0 = 2.02$ eV.

VI. SIMULATING A PURELY EXCITONIC SYSTEM WITH A PHONON BATH

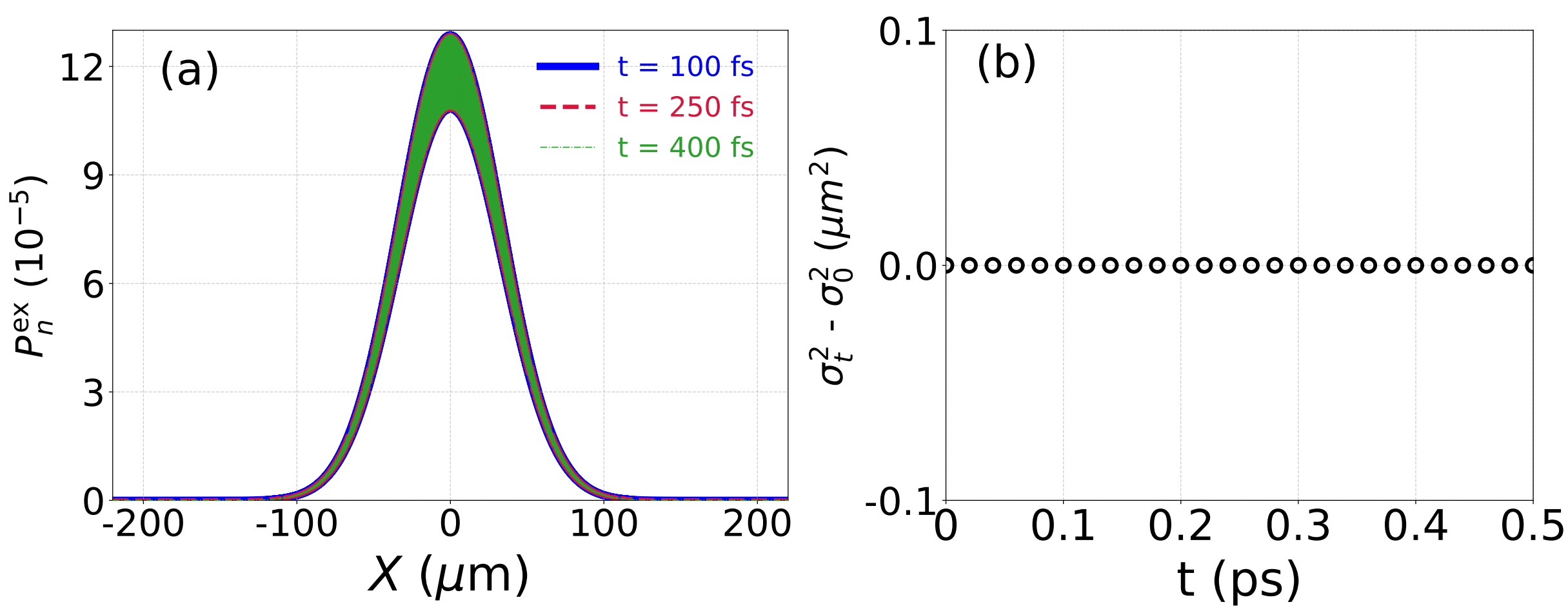

FIG. S5. Dynamics of a purely excitonic wavepacket in the absence of light–matter coupling ($g_c = 0$). (a) Site-resolved excitonic population P_n^{ex} at $t = 100$, 250, and 400 fs for an initially localized Gaussian wavepacket. The spatial profile remains essentially unchanged over time. (b) Time evolution of the mean-square displacement $\sigma_t^2 - \sigma_0^2$, remaining constant over the entire simulation window (0-0.5 ps). These results confirm the absence of intrinsic excitonic transport in our model.

In our GHTC model, the excitonic subsystem Hamiltonian is given by

$$\hat{H}_{\rm ex} = \hbar\omega_0 \sum_{n=0}^{N-1} \hat{\sigma}_n^{\dagger}\hat{\sigma}_n, \tag{S59}$$

where the exciton-exciton coupling term is absent; equivalently, the transfer integral for the excitons(τ) is intrinsically set to zero in our model. Consequently, the excitonic dispersion is completely flat and the corresponding group velocity vanishes. In the absence of a hopping term, the purely excitonic subsystem is therefore expected to be immobile when no photonic component is present.

To verify this explicitly, we performed dynamics simulations with the exciton–photon coupling switched off ($g_{\rm c} = 0$), while retaining the same exciton and phonon-bath parameters used in the polaritonic simulations. The initial state was chosen as a purely excitonic Gaussian wavepacket with a spatial width comparable to that employed in the hybrid light–matter cases.

Figure S5a shows the site-resolved excitonic population at representative times during the dynamics. The spatial profile remains nearly identical to the initial distribution, exhibiting no discernible spreading. The corresponding mean-square displacement (MSD), plotted in Fig. S5b, remains essentially constant throughout the simulation.

These results confirm that, within our model, excitons possess no intrinsic mobility in the absence of photonic coupling. Therefore, any spatial transport observed in the polaritonic simulations originates from the photonic component and its hybridization with the excitonic subsystem, rather than from exciton hopping.

---

[1] A. Mandal, M. A. Taylor, B. M. Weight, E. R. Koessler, X. Li, and P. Huo, Theoretical advances in polariton chemistry and molecular cavity quantum electrodynamics, Chem. Rev. **123**, 9786 (2023).

[2] R. H. Tichauer, J. Feist, and G. Groenhof, Multi-scale dynamics simulations of molecular polaritons: The effect of multiple cavity modes on polariton relaxation, **154**, 104112.

[3] D. Xu, A. Mandal, J. M. Baxter, S.-W. Cheng, I. Lee, H. Su, S. Liu, D. R. Reichman, and M. Delor, Ultrafast imaging of polariton propagation and interactions, Nat. Commun. **14**, 3881 (2023).

[4] R. H. Tichauer, I. Sokolovskii, and G. Groenhof, Tuning the coherent propagation of organic exciton-polaritons through the cavity q-factor, Adv. Sci. **10**, 2302650 (2023).

[5] A. Caldeira and A. Leggett, Quantum tunnelling in a dissipative system, Ann. Phys. **149**, 374 (1983).

[6] A. Nitzan, *Chemical Dynamics in Condensed Phases* (Oxford, New York, 2006).

[7] P. L. Walters, T. C. Allen, and N. Makri, Direct determination of discrete harmonic bath parameters from molecular dynamics simulations, **38**, 110.

[8] M. Balasubrahmaniyam, A. Simkhovich, A. Golombek, G. Sandik, G. Ankonina, and T. Schwartz, From enhanced diffusion to ultrafast ballistic motion of hybrid light–matter excitations, Nat. Mater. **22**, 338 (2023).

[9] T. Sfez, E. Descrovi, L. Yu, M. Quaglio, L. Dominici, W. Nakagawa, F. Michelotti, F. Giorgis, and H. P. Herzig, Two-dimensional optics on silicon nitride multilayer: Refraction of bloch surface waves, Applied Physics Letters **96**, 151101 (2010).

[10] I. Sokolovskii, Y. Luo, and G. Groenhof, Disentangling enhanced diffusion and ballistic motion of excitons coupled to bloch surface waves with molecular dynamics simulations, **16**, 6719.

[11] M. Amin, E. R. Koessler, O. Morshed, F. Awan, N. M. B. Cogan, R. Collison, T. M. Tumiel, W. Girten, C. Leiter, A. N. Vamivakas, P. Huo, and T. D. Krauss, Cavity controlled upconversion in cdse nanoplatelet polaritons, ACS Nano **18**, 21388 (2024).

[12] B. X. K. Chng, M. E. Mondal, W. Ying, and P. Huo, Quantum dynamics simulations of exciton polariton transport, Nano Lett. **25**, 1617 (2025).

[13] L. Qiu, A. Mandal, O. Morshed, M. T. Meidenbauer, W. Girten, P. Huo, A. N. Vamivakas, and T. D. Krauss, Molecular polaritons generated from strong coupling between cdse nanoplatelets and a dielectric optical cavity, J. Phys. Chem. Lett. **12**, 5030 (2021).

[14] H. Deng, H. Haug, and Y. Yamamoto, Exciton-polariton bose-einstein condensation, Rev. Mod. Phys. **82**, 1489 (2010).

[15] Y. Hong, D. Xu, and M. Delor, Exciton delocalization suppresses polariton scattering **0**, 10.1016/j.chempr.2025.102759.

[16] M. E. Mondal, A. N. Vamivakas, S. T. Cundiff, T. D. Krauss, and P. Huo, Polariton spectra under the collective coupling regime. i. efficient simulation of linear spectra and quantum dynamics, The Journal of Chemical Physics **162**, 014114 (2025).

[17] M. E. Mondal, A. N. Vamivakas, S. T. Cundiff, T. D. Krauss, and P. Huo, Polariton spectra under the collective coupling regime. ii. 2d non-linear spectra, The Journal of Chemical Physics **162** (2025).

[18] O. Voronycha, A. Buraczewskia, M. Matuszewski, and M. Stobińsk, Numerical modeling of exciton–polariton bose–einstein condensate in a microcavity, Comp. Phys. Comm. **215**, 246–258 (2017).
